# Gapless genome assembly of *Colletotrichum higginsianum* reveals chromosome structure and association of transposable elements with secondary metabolite gene clusters

**DOI:** 10.1186/s12864-017-4083-x

**Published:** 2017-08-29

**Authors:** Jean-Félix Dallery, Nicolas Lapalu, Antonios Zampounis, Sandrine Pigné, Isabelle Luyten, Joëlle Amselem, Alexander H. J. Wittenberg, Shiguo Zhou, Marisa V. de Queiroz, Guillaume P. Robin, Annie Auger, Matthieu Hainaut, Bernard Henrissat, Ki-Tae Kim, Yong-Hwan Lee, Olivier Lespinet, David C. Schwartz, Michael R. Thon, Richard J. O’Connell

**Affiliations:** 1 UMR BIOGER, INRA, AgroParisTech, Université Paris-Saclay, Thiverval-Grignon, France; 2 Present Address: Department of Deciduous Fruit Trees, Institute of Plant Breeding and Plant Genetic Resources, Hellenic Agricultural Organization ‘Demeter’, Naoussa, Greece; 3 UR1164 URGI, INRA, Versailles, France; 4 KeyGene N.V., Wageningen, The Netherlands; 5 Laboratory for Molecular and Computational Genomics, Department of Chemistry, Laboratory of Genetics, University of Wisconsin-Madison, Madison, Wisconsin USA; 6 Laboratório de Genética Molecular de Fungos, Universidade Federal de Viçosa, Viçosa, Brazil; 7 CNRS UMR 7257, Aix-Marseille University, Marseille, France; 8 INRA, USC 1408 AFMB, Marseille, France; 9 Department of Biological Sciences, King Abdulaziz University, Jeddah, Saudi Arabia; 10 Department of Agricultural Biotechnology, Center for Fungal Genetic Resources, Seoul National University, Seoul, Korea; 11 Laboratoire de Recherche en Informatique, CNRS, Université Paris-Sud, Orsay, France; 12 Institute for Integrative Biology of the Cell (I2BC), CEA, CNRS, Université Paris-Sud, Orsay, France; 13 Instituto Hispano-Luso de Investigaciones Agrarias (CIALE), Department of Microbiology and Genetics, University of Salamanca, Salamanca, Spain

**Keywords:** Fungal genome, SMRT sequencing, optical map, transposable elements, secondary metabolism genes, subtelomeres, segmental duplication, accessory chromosomes, *Colletotrichum higginsianum*

## Abstract

**Background:**

The ascomycete fungus *Colletotrichum higginsianum* causes anthracnose disease of brassica crops and the model plant *Arabidopsis thaliana*. Previous versions of the genome sequence were highly fragmented, causing errors in the prediction of protein-coding genes and preventing the analysis of repetitive sequences and genome architecture.

**Results:**

Here, we re-sequenced the genome using single-molecule real-time (SMRT) sequencing technology and, in combination with optical map data, this provided a gapless assembly of all twelve chromosomes except for the ribosomal DNA repeat cluster on chromosome 7. The more accurate gene annotation made possible by this new assembly revealed a large repertoire of secondary metabolism (SM) key genes (89) and putative biosynthetic pathways (77 SM gene clusters). The two mini-chromosomes differed from the ten core chromosomes in being repeat- and AT-rich and gene-poor but were significantly enriched with genes encoding putative secreted effector proteins. Transposable elements (TEs) were found to occupy 7% of the genome by length. Certain TE families showed a statistically significant association with effector genes and SM cluster genes and were transcriptionally active at particular stages of fungal development. All 24 subtelomeres were found to contain one of three highly-conserved repeat elements which, by providing sites for homologous recombination, were probably instrumental in four segmental duplications.

**Conclusion:**

The gapless genome of *C. higginsianum* provides access to repeat-rich regions that were previously poorly assembled, notably the mini-chromosomes and subtelomeres, and allowed prediction of the complete SM gene repertoire. It also provides insights into the potential role of TEs in gene and genome evolution and host adaptation in this asexual pathogen.

**Electronic supplementary material:**

The online version of this article (10.1186/s12864-017-4083-x) contains supplementary material, which is available to authorized users.

## Background

Thousands of fungal genome sequences, covering 996 different species, are currently available in public databases (April 2017, http://www.ncbi.nlm.nih.gov/genome/browse/). The majority of these comprise fragmented draft assemblies that were produced from relatively short DNA sequence reads generated by the Sanger method (up to 1,000 bp) or ‘second-generation sequencing’ (SGS) technology such as 454 and Illumina (up to 500 bp) [1]. Although fragmented genome assemblies can provide good coverage of the ‘gene space’, corresponding to the protein-coding genes, in-depth studies of genome architecture, evolution and speciation of organisms require access to other parts of the genome which until recently were regarded as ‘junk’ DNA, for example regions containing repetitive sequences such as transposable elements [2]. During *de novo* genome assembly, repeats that are longer than the sequence reads create gaps, and in addition identical repeats may be collapsed on to each other or misassembled [3]. Third-generation sequencing (TGS) methods such as single-molecule real-time (SMRT) sequencing and Nanopore sequencing produce reads up to 60 kb in length that are potentially long enough to span repetitive regions. Thus, TGS methods open the possibility to obtain complete genome assemblies, either using hybrid approaches where TGS is used for completeness and SGS for sequencing accuracy [4, 5], or by combining TGS with optical mapping [6–8], which provides high-resolution restriction maps to assist assembly editing and to assign sequence contigs to chromosomes [9].

*Colletotrichum higginsianum* is responsible for severe yield losses on brassica crops in tropical and subtropical regions [10–12]. In addition to cultivated *Brassica* and *Raphanus* species it also infects the model plant *Arabidopsis thaliana*, providing a tractable model pathosystem in which both partners can be genetically manipulated. Based on optical mapping, we previously reported that the genome of *C. higginsianum* strain IMI 349063 comprises 12 chromosomes including two minichromosomes <1 Mb in length [13], consistent with results obtained more recently from mitotic cytological karyotyping [14]. This strain was sequenced in 2009 using a combination of short-read data from 454 GS-FLX (350 bp) and Illumina GAII (100 bp) sequencing platforms together with a small quantity of Sanger reads (Table 1). The resulting assembly (GenBank accession number CACQ02000000) was highly fragmented, containing 10,269 small contigs (N50 = 6,150 bp) and 376 scaffolds [13]. One limitation of this assembly was that many of the predicted 16,172 protein-coding genes were truncated (9%) or split between contigs, resulting in multiple gene calls (7%). Fragmentation was especially problematic for the prediction of large secondary metabolism key genes and gene clusters. In addition, transposable elements could not be annotated because repetitive sequences had been largely eliminated during assembly. Furthermore, few of the sequence scaffolds were large enough to be unambiguously aligned to the optical map, so that chromosome locations could not be ascribed to genes or repeat elements.

**Table 1:** Comparison of *Colletotrichum higginsianum* genome assemblies and annotations

Input data & Assembly statistics	NCBI accession number	
	CACQ02000000	LTAN01000000
Type of input data:		
PacBio P5-C3 read coverage	-	133x
Sanger Fosmid (For/Rev) read coverage	0.2x	-
Illumina GAII read coverage	76x	-
454 GS-FLX Titanium read coverage	25x	-
Chromosome number^a^	12	12
Genome physical size^b^	53.35 Mb	53.35 Mb
Assembly length	49.08 Mb	50.72 Mb
Total sequence alignable to optical map	77.14 kb	50.38 Mb
Number of contigs	10,269	28
Largest contig	49.23 kb	6.04 Mb
N50 contig length	6.15 kb	5.20 Mb
G+C content	55.10%	51.86%
Coverage by Transposable Elements^c^	1.2%	7.0%
Coverage by Simple Sequence Repeats^d^	-	12.7%
Number of predicted gene models^e^	16,172	14,651
Genes with RNA-Seq evidence^f^	14,502	12,878
Annotation completeness (BUSCO)^g^		
Complete genes	2,946 (79%)	3,616 (97%)
Fragmented genes	569 (15%)	76 (2%)
Missing genes	210 (6%)	33 (0.9%)

^a^Independently determined by optical mapping [13] and cytological karyotyping [14]

^b^Estimated by optical mapping

^c^TEs were detected using RepeatMasker for assembly CACQ02000000 and REPET for assembly LTAN01000000

^d^SSRs were detected using REPET for assembly LTAN01000000 (not analyzed for assembly CACQ02000000)

^e^Different gene annotation pipelines were used for each assembly

^f^Five or more mapped Illumina reads

^g^Gene annotation completeness was estimated using a set of 3,725 Sordariomycete Benchmarking Universal Single-Copy Orthologs (BUSCOs)

Here, we re-sequenced the same *C. higginsianum* strain using Pacific Biosciences SMRT sequencing to make a *de novo* assembly. By combining this with previous optical mapping data, we obtained a near-complete assembly of the nuclear genome, in which all 12 chromosomes are sequenced telomere to telomere with no gaps, except for one region containing the rDNA repeats. The gapless assembly enabled a more precise annotation of protein-coding genes in *C. higginsianum* and allowed us to obtain a comprehensive inventory of secondary metabolism-related genes and gene clusters, many of which are new. Genomic regions that were previously badly assembled are now accessible to analysis, notably the two mini-chromosomes, which are revealed to differ markedly from the core genome in their gene and repeat content. An accurate annotation of repeats uncovered a significant association of TEs, including some that are transcriptionally active, with genes encoding secreted effector proteins and secondary metabolism genes. Finally, the complete assembly enabled us to identify chromosome segmental duplications associated with highly conserved subtelomeric TEs, which provide potential sites for homologous recombination.

## Methods

### Genome sequencing and assembly

High molecular-weight genomic DNA was extracted from mycelium of 3 day-old liquid cultures of *C. higginsianum* strain IMI 349063 as follows. After grinding the mycelium in liquid nitrogen with a mortar and pestle, DNA was extracted using Nucleobond AXG100 columns (Macherey Nagel, Ref. 740545) according to the manufacturer’s instructions. Approximately 10 μg of genomic DNA was used to prepare a ~20 Kb size-selected library and then sequenced on the Pacific Biosciences RS II platform at Keygene N.V., Wageningen, The Netherlands using the P5-C3 polymerase-chemistry combination and 240 min movie time. For *de novo* assembly of the sequence data we used the Hierarchical Genome Assembly Process (HGAP) approach [15] (SMRT analysis version 2.3.0, HGAP3.0). Reads were first filtered (minimum read length = 500 bp; minimum read quality = 0.8; minimum sub-read length = 500 bp) and then long, highly accurate sequences were pre-assembled by mapping the single-pass reads onto longer ‘seed’ reads. The Overlap Layout Consensus algorithm (WGS-Celera Assembler 7.0) was then used to perform an HGAP assembly of the pre-assembled reads. Finally, InDel and base substitution errors remaining in the draft assembly were reduced by polishing the consensus sequence using Quiver (https://github.com/PacificBiosciences/GenomicConsensus). The assembly was validated by Polymerase Chain Reaction (PCR) with primers shown in Additional file 1 using standard molecular biology techniques [16]. For Illumina sequencing, libraries were prepared from 1 μg of genomic DNA at the Max Planck Genome Centre Cologne and sequenced on the Illumina Hiseq 2500 platform to produce 100 bp paired-end reads. These data were used exclusively for detecting sequence polymorphisms and were not included in the genome assembly.

### Genome assembly comparison and sequence accuracy

Whole-genome alignments between contigs of the old and new genome assemblies were performed using MUMmer 3.0 [17]. To assess the accuracy of the PacBio-derived genome sequence, we mapped Illumina paired-end 100 bp reads to the new assembly using BWA-MEM v. 0.7.15 [18]. After filtering to retain only uniquely mapped and properly paired reads, Freebayes [19] was used to detect sequence polymorphisms (SNPs, Indels) between the Illumina reads and the PacBio assembly. To limit false-positive detections, two filters were applied sequentially, as recommended for whole-genome variant calling [20] using the VCFFiltering script (https://urgi.versailles.inra.fr/download/gandalf/VCFtools-1.2.tar.gz). The first-pass filter eliminated variants located in low complexity regions detected by mdust [21] or in annotated TEs (AN <2, AF >0.9, 98<DP>201). In the second pass, parameters were relaxed to allow detection of weakly covered variants (AN <2, AF <0.8, 12<DP).

### Detection and annotation of transposable elements and simple sequence repeats

Two pipelines from the REPET package (http://urgi.versailles.inra.fr/tools/REPET) were used to annotate transposable elements (TEs). The TEdenovo pipeline [22] was used to detect repeats in the genome, build consensus sequences and to classify them [23]. Consensus sequences classified as simple sequence repeats (SSR) or those built from less than 10 ‘high-scoring segment pairs’ were filtered out. The remaining 91 consensus sequences were added to 11 TE sequences previously reported from other *Colletotrichum* species (Additional file 2). The library of 102 consensus sequences was used to annotate TE copies in the genome using the TEannot pipeline [22]. The results were manually filtered for consensus sequences lacking a full-length copy in the genome, chimeric sequences and potential host genes. TEannot was then run again using the new library of 41 filtered consensus sequences. Multiple alignments of full-length copies from each TE family against the genome assembly were performed using Muscle v3.8.31 [24].

### Analysis of Repeat-Induced Point mutation (RIP)

Phylogenetic trees for DNA methyl transferases were built as described previously [25] with PhyML [26] from a multiple alignment generated with T-Coffee [27] and manually edited to remove non-informative sites. Sequence divergence plots were drawn as described by Maumus et al. [28]. RIP analyses followed the steps described previously [25]. Briefly, (i) each TE copy was aligned against its consensus in a pair-wise manner with REPET tools (refalign, refalign2fasta) to derive a multiple alignment, (ii) TE copies smaller than 400 bp and with less than 80 % identity with the consensus were filtered out, (iii) RIPCAL [29] was applied to each TE family, using the copy with highest GC content to compute base transition, and (iv) in-house Perl and R scripts were used to calculate dinucleotide bias and produce graphical outputs. To increase the weak RIP signal observed for RLX families, we relaxed the threshold Ti/Tv ratio to 1.5, instead of 2.0 as more commonly used.

### Gene prediction

Coding genes were predicted using the MAKER2 pipeline, version 2.31.8 [30]. The SNAP *ab initio* gene finder [31] was trained with protein homology evidence from 47,455 fungal annotated genes in the UniProt database (http://www.uniprot.org/; release 2015_08) and 16,172 predicted genes from the previous *C. higginsianum* annotation (http://fungi.ensembl.org/Colletotrichum_higginsianum/Info/Index), and transcriptomic data derived from Sanger and 454 GS FLEX ESTs [32], and Illumina RNA-seq reads [13]. For the latter, we made a genome-guided transcript assembly by mapping the Illumina reads to the genome sequence with TopHat2 [33] followed by assembly with Cufflinks v.2.2.1 [34]. MAKER2 was run with the SNAP models and the resulting gene models were used to train Augustus 3.1.0 [35]. MAKER2 was then run a second time using the trained files from SNAP and Augustus (*Fusarium graminearum*) as well as the *de novo* predicted gene models from Augustus. Some gene structures were inspected and manually corrected where necessary using Geneious version R8 [36]. To compare gene content between the new and old annotations of *C. higginsianum*, we used BUSCO v.1.2 to search for a set of 3,725 Sordariomycete universal single-copy orthologous genes [37]. In addition, we aligned the 16,172 CDS predicted in the old annotation against the new genome assembly using Blat [38]. After filtering with pslReps, the results were exported with pslToBed into BEDtools [39] to find the correspondence between the old and new CDS predictions.

### Functional annotation

Functional annotations for the predicted proteins were obtained using BLASTP to search the UniProt/SwissProt protein database and Blast2GO. The Fungal Transcription Factor Database [40] and Fungal Cytochrome P450 Database [41] were used to annotate transcription factors and cytochrome P450 enzymes, respectively. The CAZy annotation pipeline ([42], http://www.cazy.org) was used to annotate the repertoire of carbohydrate-active enzymes. Secondary metabolism key enzyme-encoding genes (SMKGs) and gene clusters were identified by combining predictions from SMURF [43], antiSMASH v.3.0 [44], SMIPS [45], CASSIS [45] and an in-house pipeline reported previously [46]. Clusters were further defined based on gene co-expression [47]. Extracellular secreted proteins (with no predicted transmembrane domain or a GPI-anchor) were predicted using SignalP v.4.1 [48] and PredGPI [49]. Candidate Secreted Effector Proteins (CSEPs) were defined as extracellular secreted proteins that were not present in species outside the genus *Colletotrichum*, based on BLAST searches against the NCBI nr database (27.07.2016). We further categorised the CSEPs as either genus- or species-specific based on the BLAST results. To identify secreted proteases, sequences of extracellular proteins were subjected to batch BLAST against the MEROPS database [50].

### Phylogenetic analysis of secondary metabolism key genes

Concatenated sequences of the KS and AT domains of predicted *C. higginsianum* PKS and PKS-NRPS hybrids were aligned with well-characterized enzymes experimentally linked to a metabolite using Muscle [24]. Further sequences from well-annotated fungal genomes were included in the dataset (Additional file 3). Evolutionary history was inferred using the Maximum Likelihood method based on the Le and Gascuel model with 1,000 iterations [51]. The initial tree for the heuristic search was obtained by applying Neighbor-Join and BioNJ algorithms to a matrix of pairwise distances estimated using a JTT model, and then selecting the topology with superior log likelihood value. A discrete Gamma distribution was used to model evolutionary rate differences among sites (gamma parameter, 4). Less than 30 % of alignment gaps, missing data, and ambiguous bases were allowed at any one position. Evolutionary analyses were conducted in MEGA6 [52] and the tree was edited with Treedyn version 198.3 [53].

### Relationship of TEs to genes and gene clusters

The distances between TEs and (a) genes encoding candidate secreted effector proteins, and (b) all genes contained within secondary metabolism gene clusters were analysed using permutation tests implemented in the R package regioneR [54]. The mean distance between each gene in each functional category and the nearest TE was compared to the mean distance of a random sample of genes taken from the whole genome. Ten thousand random permutations were sampled from the whole genome to establish a distribution of means, which was then used to calculate a p-value for each gene class.

### Segmental duplication analysis

To detect segmental duplications (SDs) we developed a new tool called SDDetector (https://github.com/nlapalu/SDDetector), based on the protocol of Khaja *et al*. [55]. Using this tool, we performed a soft-masked megablast (version 2.3.0+) alignment of the PacBio unitigs to the TE-masked genome. Matches were then chained together based on the following parameters: minimum sequence identity = 90%, maximum gap size between fragments = 3 kb, minimum fragment size = 3 kb. SD gene content was analysed to detect sequence polymorphisms among duplicated genes, and their potential effects were manually inspected. Genomic regions (≥ 5 kb) bordering each SD were extracted and compared to the TE annotation. In cases of overlap with TEs, extracted regions were extended up to the end of TE features and corresponding sequences involved in the SD were subjected to Blast in a pair-wise manner. A sequence identity of 80% and the length of Blast matches were used as criteria to evaluate SD border sequence similarity and the possible role of TEs in duplication events.

### Transcriptome analysis

For gene expression profiling, we re-analysed previously published RNA-Seq data [13], corresponding to four developmental stages of *C. higginsianum*, namely appressoria in vitro (22 hpi), appressoria *in planta* (22 hpi), the biotrophic stage of infection (40 hpi), and the switch from biotrophy to necrotrophy (60 hpi). These data sets comprising 100 bp single reads (3 replicates per stage) are available under GEO accession number GSE33683. Filtered reads were mapped with TopHat2 [33] (version 2.0.14, I = 5000, a = 10, g = 5) against the new annotation of the *C. higginsianum* genome. HTseq [56] (version 0.5.3p9) was used to count reads per gene before statistical analysis with DESeq2 version 1.1.0 [57] using default parameters. Genes were considered differentially expressed if |log2 FC| ≥ 2, q-value < 0.01. To evaluate TE expression, we used three of the above-mentioned RNA-Seq data sets, namely appressoria in vitro, appressoria *in planta* and the biotrophic phase. After read mapping with TopHat2, read counts were obtained for TEs using FeatureCounts [58] with or without the option ‘multi-mapped reads’ (-M). Counts were then transformed into average log(*CPM*_*i*_) according to the formula below, where *i* = total number of TE copies, *n* = number of replicates, *N* = number of mapped reads, *X* = (read counts +1).

$$ av erage\ \mathit{\log}\ \left({CPM}_i\right)=\frac{1}{n}{\sum}_{j=1}^n\left(\mathit{\log}\left(\frac{X_{ij}}{N_j}.{10}^6\right)\right) $$

Expressed and non-expressed TE copies were discriminated according to their log (CPM) distribution. Expressed TEs in at least one condition were clustered by k-means using R scripts.

### Generation of a fungal reporter strain

For constructing a transcriptional reporter strain, the promoter of polyketide synthase gene *ChPKS38* (CH63R_14350) was fused to the red fluorescent protein gene *mRFP*. The 1.3kb promoter of *ChPKS38* was amplified with Phusion polymerase (ThermoFisher Scientific, Waltham, Massachusetts) using primer pair 1 and the 1.2kb 3' flanking region of *ChPKS38* with primer pair 2. The mRFP and G418 resistance genes were amplified from the plasmid pFPL-Rg [59] with primer pairs 3 and 4, respectively. The four fragments were then joined using primer pair 5 by double-joint PCR [60]. All primers are listed in Additional file 1. The resulting cassette was cloned into the pCR-BluntII-TOPO vector (450245, Invitrogen, Carlsbad, California) to give the plasmid pCRII-pChPKS38RFP used for fungal transformation.

### Fungal transformation

The *C. higginsianum* wild-type strain was used for PEG-mediated transformation of protoplasts. Spores were germinated in liquid Mathur's medium for 16-18h, harvested by filtration and resuspended in digestion mix (0.7 M NaCl; 1 M NaPO_4_, pH 5.8; 30 mg.ml^-1^ lysing enzyme (Sigma L1412, St-Louis, Missouri), pH adjusted to 5.6). After digestion for 3-4 h at 25°C with gentle shaking, the protoplasts were filtered through 30 μm nylon mesh, washed twice each with cold 0.7 M NaCl and cold STC (1.2 M Sorbitol, 10 mM Tris-HCl pH 7.5, 50 mM CaCl_2_), and either used immediately or stored at -80°C. For transformation, 10^7^ protoplasts were incubated on ice with 5-10 μg of DNA for 20min. After adding successively 1 volume, 1 volume and 4 volumes of PEG solution (60% w/v PEG4000, 10 mM Tris-HCl pH 7.5, 50 mM CaCl_2_) followed by 1 mL STC, the protoplasts were plated with regeneration medium (1M sucrose, 0.1%, yeast extract, 0.1% casein hydrolysate, 1.6% agar). After growth for 16 h at 25°C, the regeneration medium was over-layered with 1% agar containing 300 μg.ml^-1^ G418 (geneticin). Transformants were selected on PDA supplemented with 300 μg.ml^-1^ G418 and screened by fluorescence microscopy.

### Confocal microscopy

Spores of the *pChPKS38::mRFP* reporter strain were inoculated either onto dialysis tubing (Visking, Roth) or the cotyledons of 7-day old seedlings of *Arabidopsis thaliana* Col-0 as described previously [32]. Images of mRFP fluorescence (excitation: 532 nm; emission: 588-621 nm) were recorded using a Leica SPE confocal microscope with a 63x/NA 1.15 water-immersion objective. Images were analysed using Fiji software and the FigureJ plugin [61, 62].

## Results

### Genome sequencing and assembly

**Fig 1:**
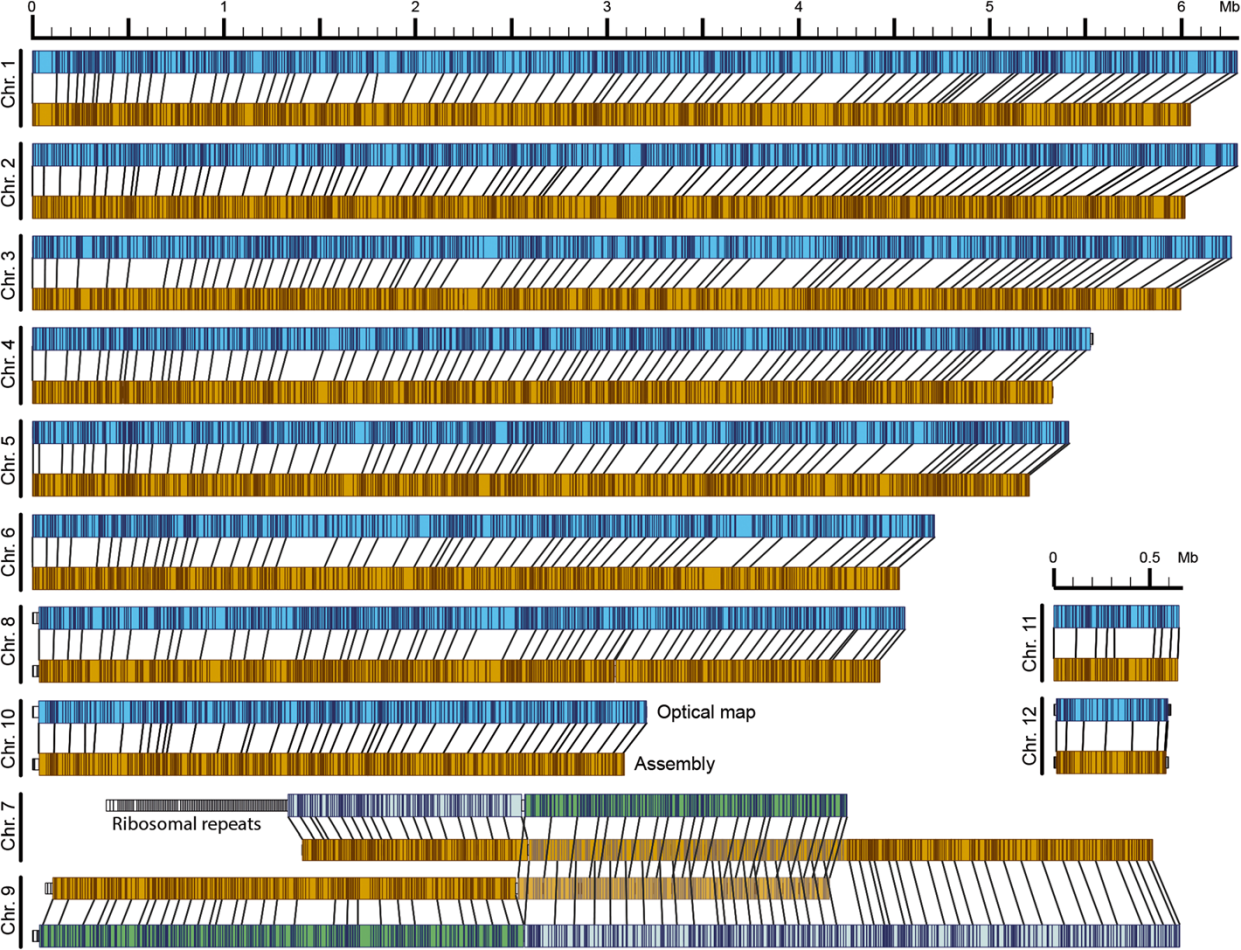
Validation of the *C. higginsianum* genome assembly by alignment of unitig sequences (orange) against chromosome optical maps (blue). MluI restriction sites are represented in optical maps and unitigs by vertical bars. Chromosomes 7 and 9 show discrepancies between unitigs and optical maps. These optical maps are colour-coded to highlight the break-points

Sequencing a total of 15 SMRT cells produced 7.8 Gb of raw sequence reads, and after quality and length filtering, the remaining reads provided ~133× genome coverage. A total of 92,834 error-corrected reads (N50 length = 16,193 bp) were assembled using the Celera Assembler to give a raw assembly of 44 unitigs. These were then aligned to the *C. higginsianum* optical map [13] using map aligner software to order and orientate the contigs and to identify overlaps between them. The 16 largest unitigs aligned to the chromosome optical maps while the remainder were too small (17.0-34.8 kb) to be unambiguously mapped. Overlapping contigs were merged and validated by re-alignment to the optical map using stringent settings (Fig. 1). The final edited assembly contained 28 unitigs with a total length of 50.82 Mb (Additional file 4). The 12 largest unitigs correspond to the expected number of chromosomes and account for 99.14% of the total genome assembly. Eleven of the 12 chromosomes are completely sequenced from telomere to telomere without gaps. Only the 5’ region of chromosome 7, corresponding to the rDNA repeats, remains incompletely assembled and is represented by 13 small unitigs. A further three unitigs contain the mitochondrial genome.

Two striking breaks in the alignment concern chromosomes 7 and 9 (Fig. 1). PCR using primer pairs located on each side of the putative break-points confirmed the sequence continuity of unitigs 7 and 9 and did not support the optical map (Additional file 5A). However, the chromosome optical maps show no evidence for misassembly of the raw single DNA molecule maps at these break-points. One possible explanation for this anomaly is that a chromosome translocation event occurred after the strain was subjected to optical mapping in 2010 and before the genome was re-sequenced in 2015.

The consensus calling results obtained from Quiver indicated that the sequence accuracy of this assembly is high (≥99.9%). To verify this, we mapped Illumina 100 bp paired-end reads (total 9.07 Gb, ~178× genome coverage) against the assembly. After filtering to exclude false positives, only 21 InDels and no SNPs were detected with strict filtering, while with less stringent parameters, 87 InDels and 11 weakly-covered SNPs were recovered. InDels predominantly affected nucleotides within tracts of homopolymer sequence, as noted previously for SMRT sequencing data [63].

### Comparing old and new gene annotations

A total of 14,651 protein-coding gene models were predicted by the MAKER2 pipeline from the new genome assembly, 1521 fewer than were predicted in the previous annotation (Table 1) [13]. Although different gene-calling pipelines were used, this discrepancy largely reflects the reduced number of fragmented and truncated genes in the new assembly. Thus, among 3,725 Sordariomycete Benchmarking Universal Single-Copy Orthologous genes [37] the proportion classified as ‘fragmented’ declined from 15% in the old annotation to 2% in the new annotation, and the proportion classified as ‘missing’ reduced from 6% to 0.9% (Table 1). Further evidence that the gapless assembly has resolved the problem of split gene models came from aligning the old gene annotation to the new assembly, revealing that 2,699 MAKER2 genes match to two or more of the previous gene models. The new annotation includes 2,289 new genes with no match in the previous annotation. The majority of these were not previously predicted due to their fragmentation between contigs or because transcript evidence from RNA-Seq was not used in the previous annotation. The new genes include all but one of the 133 genes on chromosome 12, which was covered by only two small contigs in the old assembly (Additional file 6). Conversely, 944 genes from the old annotation are absent from the new annotation, most of which correspond to putative ORFs inside TEs or transposases and reverse transcriptases that were excluded by the MAKER2 pipeline. The correspondence between old and new gene IDs is shown in Additional file 7. Taken together, these data indicate the quality of the revised annotation is dramatically improved compared to the previous version, largely due to the absence of gaps in the new assembly.

Based on the new gene annotation, we re-predicted genes encoding transcription factors, cytochrome P450 enzymes, carbohydrate-active enzymes, secreted proteins, candidate secreted effector proteins (CSEPs) and secreted proteases. A detailed comparison of the new and old annotations of these gene categories is beyond the scope of the present paper and will be reported elsewhere. Inventories of all these gene categories are provided in Additional file 8A.

### Characteristics of mini-chromosomes 11 and 12

Examination of the two mini-chromosomes showed that their gene content (~25% protein-coding genes by length) is almost 2-fold lower than that of the larger 10 ‘core’ chromosomes (mean = 46%, Table 2; Additional file 6). Moreover, a lower proportion of genes located on chromosomes 11 and 12 are expressed either in vitro or *in planta* (32 and 10%, respectively) compared to those in the core genome (mean = 54%). Conversely, chromosomes 11 and 12 are highly enriched with transposable elements (38 and 28% by length, respectively) compared to a mean of only 6% on the core chromosomes (Table 2). They are also more AT-rich (50.7% and 52.8%, respectively, compared to a mean of 45.5% for the core genome). Furthermore, the proportion of predicted genes encoding proteins of unknown function (annotated as hypothetical proteins) was 2 to 3-fold higher on the mini-chromosomes compared to the core genome (Additional file 6). Thus, nearly three-quarters of all genes on chromosome 12 encode hypothetical proteins. Interestingly, although the mini-chromosomes are not enriched with genes encoding secreted proteins relative to the core genome, they contain 2.5 to 3-fold more secreted effector genes, of which 7 are highly expressed *in planta*. In contrast, other genes potentially related to pathogenicity, e.g. those encoding secondary metabolism enzymes, CAZymes, cytochrome P450 enzymes, secreted proteases and transcription factors, are almost absent from the mini-chromosomes (Additional file 6). All the differences observed between core and mini-chromosomes were statistically significant using Fisher’s exact test (Table 2).

**Table 2:** Differences between the core chromosomes (1-10) of *Colletotrichum higginsianum* and mini-chromosomes 11 and 12

		Chromosome	
Characteristic	1-10 (mean)	11	12
Total length (bp)	4,914,036	646,208	597,935
Number of protein-coding genes	1,438	138	133
Proportion of genes by length (%)	46.0	25.5***	25.4***
Proportion of expressed genes (%)^a^	54.1	31.9**	9.8***
Number of transposable element (TE) copies	128	146	63
Proportion of TEs by length (%)	5.9	38.4***	28.0***
G+C (%)	54.5	49.3***	47.2***
Proportion of genes with unknown function (%)	25.7	55.8***	73.7***
Proportion of secreted protein genes (%)	11.2	10.1	7.5
Proportion of effector genes (%) ^b^	1.9	5.8**	4.5*

Asterisks indicate data for the mini-chromosomes differ significantly from the mean for chromosomes 1-10 (Fisher’s exact test, *** P <0.001; ** P <0.01; * P <0.05)

^a^Genes were considered to be expressed if they showed ≥1% of the expression-level of actin (corresponding to ≥10 TPM), based RNA-Seq data from one in vitro and three *in planta* samples [13]

^b^Candidate secreted effector protein genes included CSEPs predicted from the genome (secreted proteins without homologs outside the genus *Colletotrichum*) and some ChECs (*C. higginsianum* effector candidates) previously predicted from the transcriptome [32] that are absent from the new annotation or have BLAST hits to effectors from outside the genus

### Annotation of secondary metabolism genes

Large genes such as secondary metabolism key genes (SMKGs; commonly 6-8 kb, up to 37 kb) were disproportionately affected by fragmentation between multiple contigs in the old assembly, for example 23 SMKGs were fragmented into 56 separate gene models [13]. To obtain a more complete and accurate inventory, we predicted SMKGs *de novo* from the new annotation by combining predictions from SMURF [43], antiSMASH [44], SMIPS [45] and an in-house pipeline [46]. In this way, a total of 89 unique SMKGs were defined and classified into major functional categories in Table 3. Notably, 7 of the SMKGs (ChDMATS01, ChPKS27 and 40, ChTS02, 07, 09 and 14) are novel in that they have no matching gene call in the previous annotation. In addition we detected 12 NRPS-like genes (monomodular NRPSs with an unconventional reductive release domain) and one type III PKS (chalcone synthase). All SMKG predictions were manually curated and their predicted enzymatic domains are summarized in Additional file 9.

**Table 3:** Summary of predicted *C. higginsianum* secondary metabolism key genes and clusters

Gene category^a^	2012 assembly^b^	New assembly^c^
SM Clusters	47	69^d^ (8)
PKS	58	40^e^
NRPS	12	15
PKS-NRPS	6	6
TS	17	17^f^
DMATS	10	11
NRPS-like	nd	12

^a^DMATS, dimethylallyl tryptophan synthase; NRPS, non-ribosomal peptide synthetase; PKS, polyketide synthase; SM, secondary metabolism; TS, terpene synthase

^b^As published by O'Connell et al. [13]

^c^This study. Number in brackets corresponds to SM clusters with NRPS-like genes as the only key gene

^d^Includes one cluster that is duplicated with 98 % homology

^e^Two PKS genes are disrupted by TEs and one has a wrongly predicted gene model

^f^Includes one TS that is duplicated with 100% homology

**Fig. 2:**
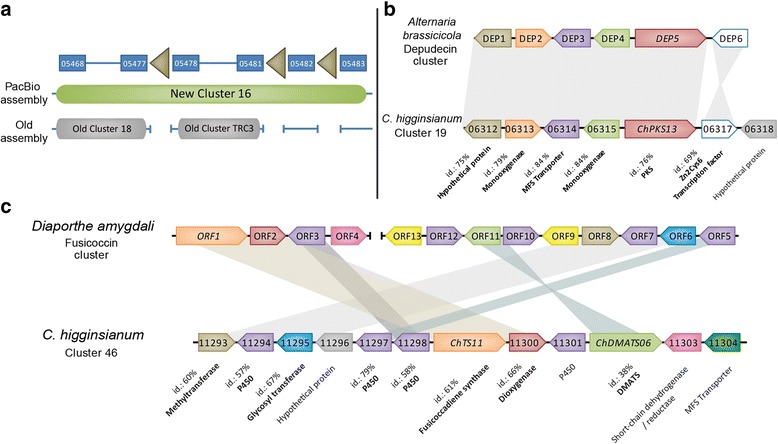
Schematic representation of selected *C. higginsianum* secondary metabolism (SM) genes clusters. **a** Resolution of a former split SM cluster by the PacBio assembly. The new cluster 16 encompasses four contigs from the old assembly [13], two of which contain former clusters 18 and TRC3. Arrowheads: transposable elements **b** Comparison of cluster 19 and the depudecin cluster of *Alternaria brassicicola.* Protein identity is high (> 70%) and gene order and orientation are conserved except for the gene DEP6/CH63R_06317. **c** Comparison of cluster 46 and the fusicoccin cluster from *Diaporthe amygdali* [74]. In *D. amygdali*, genes are dispersed at two distinct loci in contrast to *C. higginsianum*. Protein identity is moderate to high and genes were extensively rearranged. Shading indicates syntenic blocks and genes pairs. Yellow: acetyl-transferase

**Fig. 3:**
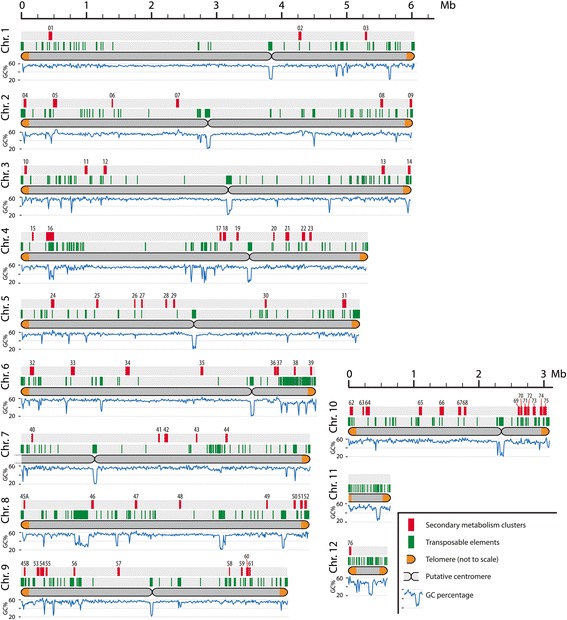
Schematic representation of the distribution of secondary metabolism gene clusters and transposable elements across the 12 *C. higginsianum* chromosomes. The 5' end of unitig_7 containing the ribosomal repeats is fragmented between 13 unitigs that are too small to align with the optical map. Putative locations of the centromeres are indicated where possible

To predict secondary metabolism (SM) gene clusters, we used a combination of SMURF [43], antiSMASH [44] and CASSIS [45], while cluster borders were further defined based on gene co-expression evidence. On this basis, 69 clusters were delimited (77 including those with only an NRPS-like key gene) compared to only 47 that were previously found [13] (Additional file 8B). Of these 77 clusters, 28 (38%) contain at least one predicted transcription factor (previously only 9). In addition to the greater number of predicted clusters, most are larger and more complete, partly because repeat-rich regions within clusters have been resolved in the new assembly. For example, new cluster 16 merges two former clusters that were separated by TE stretches (Fig. 2a). The structure and composition of all predicted gene clusters are depicted schematically in Additional file 10 together with their relationships to TEs. SM gene clusters are dispersed across most of the chromosomes but are particularly enriched on chromosome 10 (14 clusters), while there are none on chromosome 11 and only one on chromosome 12 (Fig. 3). Fifteen clusters (20%) are located within 200 kb of telomeres, and the entire terpene synthase cluster 45 is duplicated between the ends of chromosomes 8 and 9 due to a segmental duplication (see below).

### Expression profiling secondary metabolism genes and clusters

**Fig. 4:**
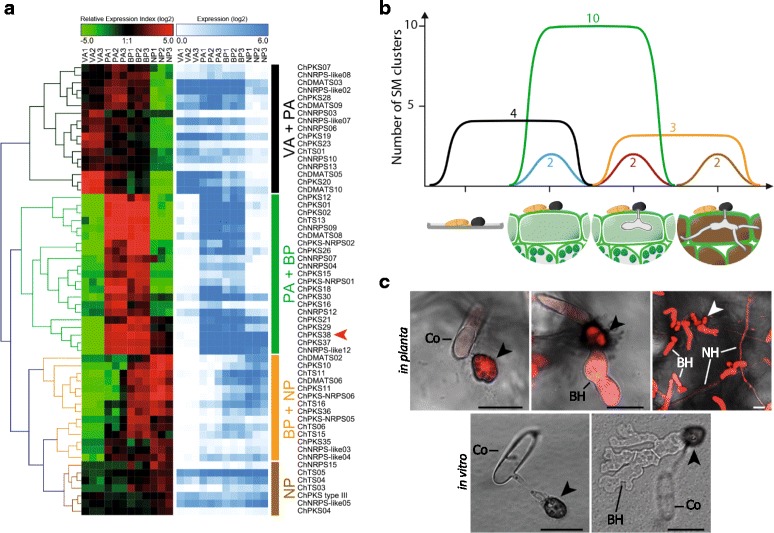
Waves of expression of secondary metabolism (SM) genes of *C. higginsianum* during infection of *Arabidopsis thaliana*. **a** Heatmap showing the expression profiles of SM key genes. Under-represented transcripts (dark green to bright green) and over-represented transcripts (dark red to bright red) are depicted as log2 relative expression index. The log2 expression levels are presented in the adjoining heatmap colour-coded from white (not expressed) to dark blue (strongly expressed). Red arrowhead: ChPKS38. **b** Schematic representation of the stage-specific expression of SM gene clusters. The expression of all genes within each cluster was evaluated using the Transcript Per Million (TPM) normalisation method. A cluster was considered expressed if more than 50% of genes had a TPM greater than 1% of the actin gene TPM, and |log2FC| ≥ 2, q-value ≤ 0.01. **c** Time-course of the expression of the pChPKS38::RFP reporter gene *in planta* and in vitro (cellophane) using confocal microscopy. All images are overlays of bright field and RFP channels captured with the same settings. RFP channels are projections of 15-25 0.2 μm optical sections. Co: conidium, arrowhead: appressorium, BH: biotrophic hypha, NH: necrotrophic hypha. Bars = 10 μm

Previous RNA-Seq datasets representing different infection stages were re-analysed (Additional file 8A) to define the expression patterns of the SMKGs. Four distinct waves of expression were recognised: (a) appressoria in vitro and *in planta*, (b) appressoria *in planta* and the biotrophic phase, (c) biotrophic and necrotrophic phases, and (d) the necrotrophic phase (Fig. 4a). Remarkably, out of the 59 significantly regulated SMKGs (|log2 FC| ≥ 2, q-value ≤ 0.01), 42 (71%) were expressed exclusively during plant infection and not in appressoria formed in vitro. To evaluate the expression patterns of entire SM gene clusters, we used the Transcript Per Million (TPM) normalisation method, where a cluster was considered to be significantly expressed if most genes in the cluster had a TPM greater than 1 % of the expression level of the actin gene, and significantly regulated if |log2FC| ≥ 2 (q-value ≤ 0.01). Among the 23 clusters expressed at any stage, more than half (14) were preferentially expressed at early stages of plant infection in appressoria and/or during the biotrophic stage, when host cells are still alive, whereas only 5 were upregulated at the switch to necrotrophy (Fig. 4b).

To examine the expression pattern of one SMKG at the cellular level, we created a transgenic reporter strain expressing the Red Fluorescent Protein (RFP) under control of the *ChPKS38* promoter. Confocal microscopy showed the reporter gene is strongly expressed (as shown by cytoplasmic RFP fluorescence) in appressoria on the plant surface before penetration, in young biotrophic hyphae formed immediately after penetration, as well as in necrotrophic hyphae (Fig. 4c). Remarkably however, no RFP fluorescence was detectable at any time-point during growth in vitro on cellophane membranes, neither in appressoria nor pseudo-biotrophic hyphae developing inside the membrane after penetration, indicating that the expression of *ChPKS38* is strictly plant-induced and not directly linked to the differentiation of specialized fungal infection structures *per se*.

### Phylogeny and comparative genomics of secondary metabolism gene clusters

Based on a phylogenetic analysis, we found that out of the 40 PKS and 6 PKS-NRPS hybrid enzymes identified in *C. higginsianum*, 12 PKS and 2 hybrids are located in clades containing a well-characterized key enzyme linked to the production of a known metabolite (Additional file 11). Using blastp we then looked for the presence of accessory genes belonging to the characterized clusters in the *C. higginsianum* proteome (Additional file 12). With this approach, we could identify 7 clusters of SM genes which are well-conserved in *C. higginsianum* and therefore likely to produce similar products (Additional file 12). These clusters correspond to Ace1 (producing a cytochalasan-related molecule) [64, 65], Alternapyrone [66], Cercosporin [67], Cytochalasin K [68], Depudecin [69], Lovastatin [70] and Melanin [13]. It is important to note that this approach can only provide a clue to the family of molecules likely to be produced rather than a definitive structure. One striking example is the cluster 19 which contains ChPKS13 and homologs of all the depudecin biosynthetic genes with 78% mean amino-acid identity (Fig. 2b). Depudecin is a histone deacetylase inhibitor produced by *Alternaria brassicicola* [69]. Based on the co-expression criterion, cluster 19 possesses an additional gene (CH63R_06318) coding for a 110 amino acid protein with no homolog in public databases and containing no functional domain that could be identified using InterProScan or the Conserved Domain Database. *C. graminicola* contains a cluster of genes including one highly reducing PKS and one non-reducing PKS that is identical in gene content and order to the RADS cluster of *Pochonia chlamydosporia* [71], which produces antifungal resorcylic acid lactones (RALs) such as monorden [72]. Interestingly, our phylogenetic analysis revealed that *C. higginsianum* encodes two HR-PKS and two NR-PKS belonging to the RALs clade with strong bootstrap support. However, the accessory genes in both *C. higginsianum* clusters (10 and 74) have a low percentage identity to those belonging to known RALs clusters, so that the final product may be significantly different (Additional files 11 and 12). It is interesting to note that the cluster 74 (containing ChPKS37 and 38) is the most highly induced SM cluster during plant infection, with peak expression during biotrophy (Fig. 4a and 4c), suggesting the product of that particular biosynthetic cluster may be crucial for establishment of the infection.

Availability of the complete sequence of chromosome 12 in the PacBio assembly allowed the discovery of a new SM cluster (cluster 76), which is located within 20 kb from a telomere and hosts four genes, including ChPKS40. The same cluster is also present in *Magnaporthe oryzae*, *Diaporthe ampelina* and *D. helianthi* with remarkably high amino-acid identity (>80%). Inference of a putative secondary metabolite was not possible for that particular cluster. Further exploration of the SM repertoire of *C. higginsianum* lead to the identification of a fusicoccadiene synthase (ChTS11). This enzyme catalyzes one of the early steps in the biosynthesis of fusicoccin A, a well-known phytotoxin which irreversibly activates plasma membrane H^+^-ATPases [73]. ChTS11 is part of a predicted cluster (cluster 46; Fig. 2c) comprising nearly all the genes described in *D. amygdali* [74]. Synteny is not conserved, probably as a result of extensive rearrangements which split the gene cluster between two different loci in *D. amygdali*. In *C. higginsianum*, cluster 46 is up-regulated at the switch to necrotrophic growth.

### Annotation of transposable elements

The content of transposable elements (TEs) in the previous genome assembly (1.2%) was significantly under-estimated due to the poor assembly of repeat-rich regions [13]. Using REPET to annotate the new gapless assembly, TEs were found to cover 7% of the *C. higginsianum* genome, while simple sequence repeats (SSRs) cover 12.7% (Table 1; Additional file 13). The TEs were classified by REPET into 41 families and named using the three-letter code of the Wicker *et al*. [75] classification (Additional file 14). The 20 families of class I retrotransposons occupy 67% of the total TE space compared to only 33% for the 20 families of class II DNA transposons (Table 4). LTR (Long terminal repeat) retrotransposons and TIR (Terminal inverted repeat) DNA transposons are the two most abundant TE orders, with 636 and 474 copies, respectively. Overall, the LTR transposon family RLX_R119 is the single most abundant TE family in the *C. higginsianum* genome, with 275 copies occupying >1 Mb by length (28% of the TE space, 42% of the retrotransposons). However, only 35 copies are complete and, taken together with the high level of divergence from the consensus sequence in this family (20.5%), the invasion of the genome by RLX_R119 was probably an ancient event. Three TE families previously described from other *Colletotrichum* species are present in the REPET annotation, namely the LTR retrotransposon CCRET1 and the non-LTR retrotransposons CCRET3 from *C. cereale* [76] and Cgt1 from *C. gloeosporioides* [77]. However, all three families are represented by few copies in *C. higginsianum*, nearly all of which are incomplete (Additional file 14). Strikingly, the 16 families of TIR transposons, mostly of the Tc1-Mariner superfamily, are represented by 4.5-fold more full-length copies than the LTR families. Moreover, we found that 13 TIR family consensus sequences, namely DTX_G154 to DTX_G164, DTX_P2.24, DTX_P21.16, DTX_P12.24, DTX_R31 and DTX_R166, contain a complete transposase gene and flanking inverted repeats and are therefore potentially active. Interestingly, in Blastn searches against the NCBI nr database the best matches to these 13 TIRs were transposases from *C. tofieldiae*, *C. incanum*, *C. sublineola* and no other *Colletotrichum* species.

**Table 4:** Major families and characteristics of transposable elements in the *C. higginsianum* genome

Type of element^a^	No. consensus^b^	No. copies	No. complete copies	Complete/incomplete copies	Genome coverage (%)^c^		TE space coverage (%)^d^	
*Class I (retrotransposons)*								
LTR	11	636	86	0.135	3.55		50.71	
LARD	2	47	10	0.213	0.67	Class I	9.57	Class I
LINE	3	50	13	0.260	0.23	4.7	3.29	67
Class I (unclassified)	4	123	2	0.016	0.24		3.43	
*Class II (DNA transposons)*								
TIR	16	474	289	0.610	1.64	Class II	23.43	Class II
MITE	1	30	17	0.567	0.04	2.3	0.57	33
Helitron	3	111	19	0.171	0.62		8.86	
*Uncategorized TEs*	1	11	4	0.364	0.01		0.14	

^a^LTR: long terminal repeat, LARD: large retrotransposon derivative element, LINE: long interspersed element, TIR: terminal inverted repeat, MITE: miniature inverted-repeat transposable element

^b^Number of TE concensus sequences in the genome

^c^Percentage of genome covered by the element

^d^Percentage of repetitive fraction covered by the element

### Identification of RIP in TE families

Repeat-induced point mutation (RIP) is a fungal-specific genome defense mechanism occurring at the pre-meiotic stage of sexual reproduction. It detects duplicated DNA sequences and induces irreversible C:G to T:A mutations at a high rate in those sequences [78]. Specific dinucleotides are often preferentially mutated, as in *Neurospora crassa* where the dinucleotide CA is the target for RIP, or *Aspergillus niger* and *A. fumigatus* where RIP occurs at CG as well as CA dinucleotides [79]. Here, we investigated the potential role of RIP in generating the striking differences in A/T content that are apparent between *C. higginsianum* TE families, which ranged from 42% in the subtelomeric family DHX_G198 up to 78% in RLX_R119, the most abundant element in the genome (Additional file 14). Using a phylogenetic analysis, we found that *C. higginsianum* possesses putative orthologues of two genes that are known to be involved in DNA methylation in other fungi (Additional file 15), namely the *RID* gene (CH63R_07391), a cytosine methyltransferase responsible for C to T mutations during RIP [80], and the *Dim-2* gene (CH63R_01196), another cytosine methyltransferase that introduces a potential bias in dinucleotide mutations [81]. Next, we searched for signatures of RIP among the copies of each TE family by looking for dinucleotide bias at sites with C/T mutations (Additional file 16A). Fifteen percent of the DTX (Class II TIR) and DHX (class II Helitron) elements display the CA dinucleotide target specific to RIP [25, 81]. In addition, 50% of the DHX elements contain the CG dinucleotide target site that could be related to the activity of Dim-2 [25, 81]. However, dinucleotide target sites for RIP mutation were not detectable among the AT-rich RLX (class I LTR) and RIX (class I LINE) elements, probably because all copies are ancient and highly mutated. To determine whether the observed RIP signatures could be correlated with the age of the TEs, we compared the relative ages of *C. higginsianum* TEs using the method of Maumus *et al.* [28]. The evolution of TE families is assumed to follow a ‘burst and decay’ model, in which identical copies proliferate and independently accumulate random mutations after integration into the genome [28]. By plotting the sequence divergence of TE copies relative to their respective family consensus sequence, which is assumed to model the intact ancestral element prior to mutation, we found evidence for a recent burst of transposition by the TIR DNA transposon (DTX) families (Additional file 16B). In contrast, the LTR retrotransposon (RLX) families showed much higher levels of sequence divergence (from 5 to 25%), consistent with more ancient transposition events. These results support our hypothesis that the LTRs are older, heavily mutated elements in which RIP is now difficult to detect using available techniques. Overall, despite the lack of evidence for sexual reproduction in *C. higginsianum*, the presence of both *RID* and *Dim-2* genes together with a signal for C to T mutations suggests that TE silencing mechanisms have contributed to restricting the invasion of this fungal genome by TEs.

### Chromosome location of TE families

**Fig. 5:**
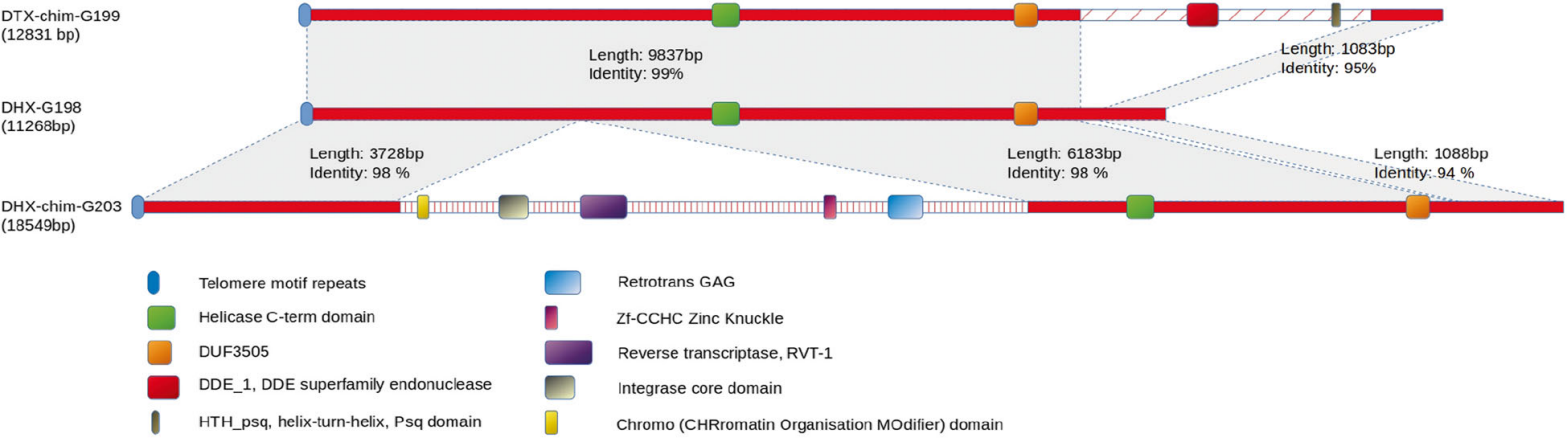
Schematic representation of the predicted domain structure of three families of conserved repeat elements present in the subtelomeric regions of all *C. higginsianum* chromosomes. DTX-chim_G199 was likely derived from DHX-G198 by the insertion of a DNA transposon, whereas DHX-chim-G203 was derived from DHX-G198 by the insertion of a non-LTR retrotransposon

Among the 41 TE families detected by REPET, 39 have at least one Full Length Copy (FLC). Analysis of the genomic location of these FLCs (Additional file 17) revealed that while two TE families are distributed across all of the 12 largest unitigs (i.e. chromosomes), others are confined to mini-chromosomes 11 and 12 (RIX_P24.14) or single chromosomes (RXX_62 and RXX_R113). The two LARD families RXX-LARD_R1 and RXX-LARD_G201 occur only on unitigs containing rDNA repeats. Notably, three TE families were detected as single FLCs at all 24 subtelomeres contained within the assembly (Additional file 18), namely DHX_G198 (12 copies) and DHX-chim_G203 (4 copies), and DTX-chim_G199 (7 copies). The number of telomere-associated copies of these families corresponded exactly to the number of Full Length Copies (FLC) detected by REPET (Additional file 14). These telomere-associated TEs are long (11.3 kb to 18.5 kb) and share large blocks of highly conserved sequence (Fig. 5). All three families encompass variable numbers of telomere repeat motifs (TTAGGG) together with predicted helicase and DUF3505 domains. In addition, DTX-chim_G199 contains DDE-1 transposase and Psq-type DNA-binding helix-turn-helix (HTH) domains, whereas DHX-chim-G203 contains five family-specific PFAM domains typical of retrotransposons, including reverse transcriptase and integrase domains (Fig. 5). Although fragmented copies of all three families occur elsewhere in the genome, FLCs occurred exclusively at subtelomeres, suggesting that FLCs are preferentially maintained at that location.

### TE expression analysis

To evaluate TE expression patterns, we used available RNA-Seq data from appressoria in vitro (VA), appressoria *in planta* (PA) and the biotrophic phase (BP). Genes (ORFs) within TEs are not annotated and no good tools are available to annotate them. Without well-defined gene models, units such as FPKM (Fragments Per Kilobase per Million mapped reads) cannot be used. We therefore calculated expression units as CPM (Counts Per Million mapped reads), either including or excluding reads mapping to multiple genomic locations. The log(CPM) distribution of multi-mapped reads showed a bimodal distribution (Additional file 19A), and we selected the central inflection point (antimode), i.e. 1.35 log(CPM), as the threshold to discriminate expressed vs non-expressed TE copies. In striking contrast, a bimodal distribution of log(CPM) was not obtained for uniquely-mapped reads (Additional file 19B). For all TE families, more expressed copies were identified with multi-mapped reads than with uniquely-mapped reads (Additional file 20). The largest number of expressed TE families (9) belonged to the TIR order of DNA transposons (DTX_G154 to DTX_G164, DTX_P12.24, DTX_R31). Interestingly, three telomere-associated TE families (DTX-chim_G199, DHX-chim_G203, DHX_G198) and two associated with the rDNA repeats (RXX-LARD_G201, RXX-LARD_R1) were also actively transcribed.

Because a quantitative differential expression analysis using multi-mapped reads is not valid, we instead performed a clustering analysis on the 441 TE copies that were expressed in at least one fungal stage. For each stage, we computed the proportion of CPM relative to the total CPM across all stages and used this Relative Index to perform *k*-means clustering (Additional file 21A). Five clusters were distinguished (Additional file 21B), from which two with contrasting profiles were selected, namely cluster 2 (high expression in VA, no expression in PA or BP) and cluster 3 (no expression in VA or PA, high expression in BP). We analysed in detail eight TE copies from these clusters showing the most extreme differential expression. All were LTR retrotransposon fragments, which in two cases comprised ‘solo’-LTRs, suggesting recombination between two LTRs lead to deletion of the internal retrotransposon sequence (Additional file 22). Among the six TE copies expressed in the biotrophic phase (cluster 3), five were located in the 3’ UTRs of genes encoding candidate effector proteins expressed at that stage, namely ChEC28, ChEC117, ChEC104 and ChEC35 and a secreted NUDIX domain protein encoded by CH63R_12509 (Additional files 21C and 22).

### Relationship of TEs to genes and gene clusters

**Fig. 6:**
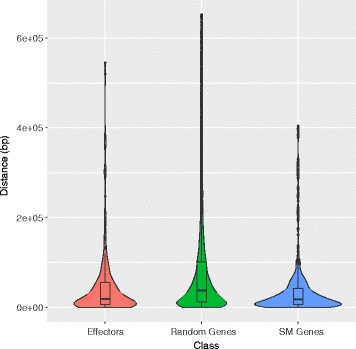
Violin plot depicting the frequency distribution of the distance (bp) between genes and the nearest transposable element (TE). The inner box plots represent the median and interquartile range of the distance for each of three gene classes. Genes located within secondary metabolism clusters (SM genes) and genes encoding candidate secreted effector proteins were located significantly closer (*p* < 0.001) to TEs than a random sample of genes taken from the genome as a whole

Manual inspection of the 77 predicted SM gene clusters revealed that 33 (43%) have at least one repetitive element located either inside the cluster or within 5 kb of the cluster border (Additional file 10). To test the statistical significance of this association, we employed a permutation test to compare the distance between TEs and genes belonging to particular functional categories. This confirmed that genes located within SM gene clusters were located significantly closer (*p* < 0.001) to TEs than a random sample of genes taken from the genome as a whole (Fig. 6). Similarly, a highly significant association (*p* < 0.001) was detected between TEs and genes encoding candidate secreted effector proteins. In addition, we found that 7 families of retrotransposons and 11 families of DNA transposons were located significantly (*p* < 0.01) closer to SM cluster genes and/or effector genes than would be expected by chance (Additional file 23). Five of these families showed a significant association with genes of both functional categories.

### Segmental duplications and their relationship to TEs

**Fig. 7:**
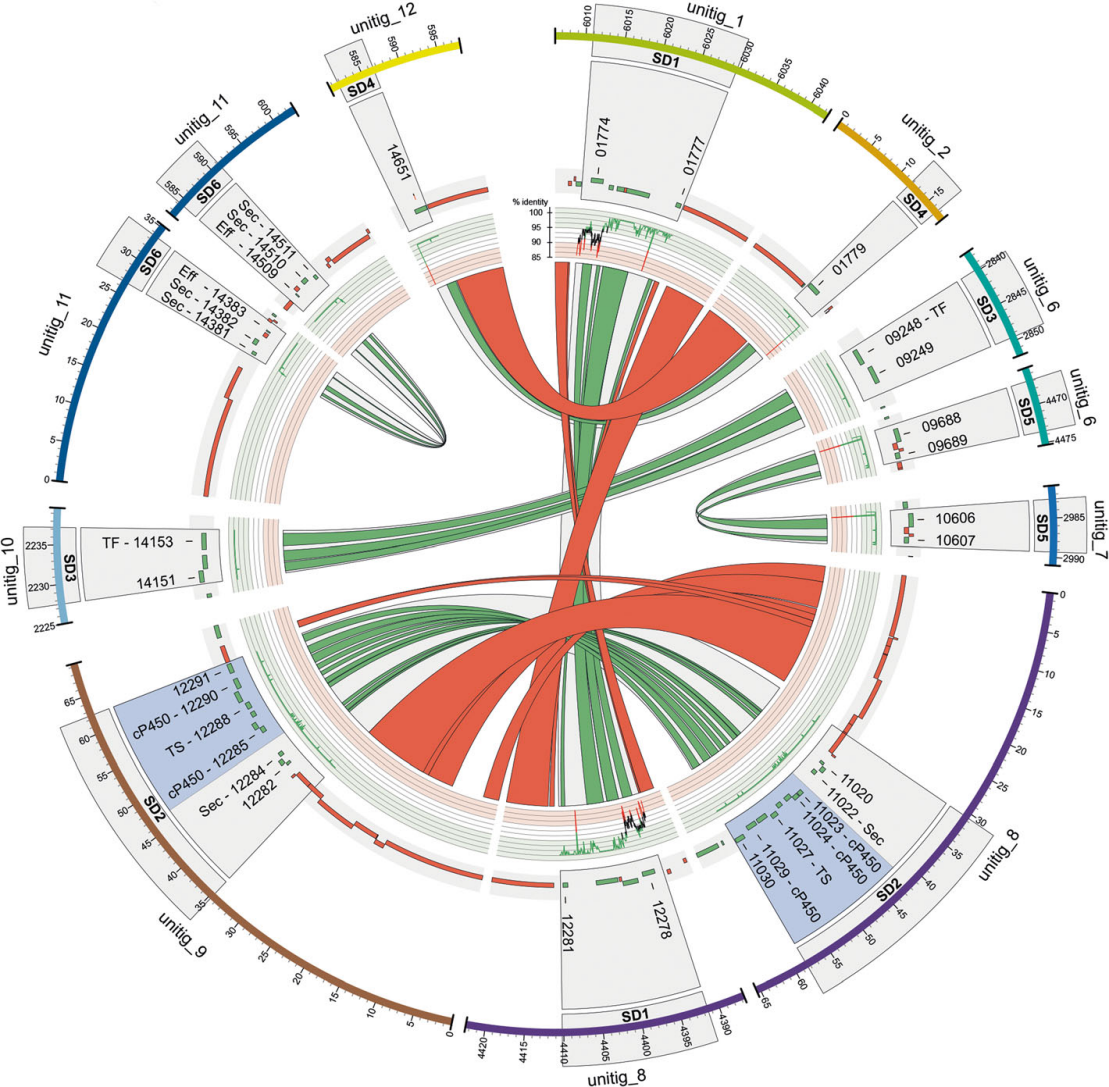
Circos plot showing segmental duplications (SDs). Genes are represented in green and transposable-elements in red. Gene IDs in each duplicated block (grey sectors) are given without the prefix "CH63R_". An entire secondary metabolism gene cluster (shaded blue) is duplicated in SD2. cP450: Cytochrome P450; Eff: Effector protein; Sec: Secreted; TF: Transcription Factor; TS: Terpene Synthase

To search for segmental duplications (SDs), we developed the SDDetector tool, based on the approach of Khaja *et al*. [55]. This revealed 11 potential duplicated regions, of which 5 were false-positives corresponding to multi-copy TE insertions. The remaining six validated SDs involve nine different chromosomes, five being inter-chromosomal duplications (SD1 to SD5) and one (SD6) intra-chromosomal (Fig. 7). SD2 was further validated by PCR (Additional file 5B). All six duplications consisted of a single alignment, suggesting that insertion/deletion of sequences has not occurred post-duplication. The duplicated regions varied in length from 4,880 bp (SD4) to 28,020 bp (SD2), with a total aligned length of ~75.4 kb (Additional file 24). Sequence polymorphism between the duplicated sequences in SD2 to SD5 was very low (0-0.18%), suggesting they result from relatively recent duplication events. In contrast, duplicated sequences in SD1 displayed a much higher level (2.6%) of mutated bases (Additional file 24), consistent with a more ancient duplication event.

A total of 46 protein-coding genes were predicted within the six duplicated regions (Additional file 25). Remarkably, SD2 involves the duplication of an entire secondary metabolism gene cluster (see TS Clusters 45A and 45B in Additional file 10) which is expressed *in planta* during biotrophy. The duplicated genes within SD6 encode three predicted secreted proteins, including effector candidate ChEC7 (CH63R_14381, CH63R_14509), which is expressed in appressoria [32]. The remaining duplicated genes either encode proteins of unknown function or do not appear pathogenicity-related. Using SDDetector to analyse polymorphisms between pairs of duplicated genes, we found all genes within SD1 are affected by numerous mutations (340 SNPs, 2 indels) producing large effects, such as amino-acid changes, introduction of premature stop codons or gene splitting (Additional file 25). The mutations show a bias favouring G->A and C->T transitions, suggesting these sequences have been subject to RIP [25]. In contrast, no mutations affecting protein sequence were detected in genes within SD4, SD5 and SD6. However, an indel in SD3 produced two gene models on unitig_10 (CH63R_14152, CH63R_14153) that correspond to only one on unitig_6 (CH63R_09249). Five indels also affected gene predictions in SD2 but all were found to be sequencing errors, possibly resulting from their location in homopolymer tracts.

Inspection of the genomic regions bordering SD3 and SD5 revealed no TE blocks or other tracts of homologous sequence. However, SD1, SD2, SD4 and SD6 were all located close to telomeres (within 30 kb), and in each case the borders contained at least one copy of a telomere-associated TE (DTX-chim_G199, DHX-chim_G203 or DHX_G198). These conserved blocks of telomere-associated TEs provide between 7.6 and 28.4 kb of homologous sequence with 88-100% identity (Additional file 26), and as such represent potential sites for homologous recombination.

## Discussion

In this paper we report the complete genome sequence and revised gene annotation for the reference strain of *C. higginsianum*, a widely-used model pathogen and member of a large genus with enormous economic impact on numerous crops worldwide. By combining the very long reads from PacBio SMRT sequencing together with optical mapping, we obtained a highly contiguous assembly, where all 12 chromosomes are sequenced telomere to telomere without gaps except for the rDNA repeats on chromosome 7. This represents the most complete genome assembly available for any *Colletotrichum* species to date and adds to only a small list of finished genomes from other phytopathogenic fungi, namely *Zymoseptoria tritici*, *Sclerotinia sclerotiorum*, *Botrytis cinerea*, *Verticillium dahliae* and *Fusarium graminearum* [6–8, 82, 83]. The new assembly has provided access to genomic regions that were previously not assembled, notably the mini-chromosomes. Importantly, the absence of gaps in the assembly enabled a much more accurate annotation of both protein-coding genes and repetitive elements in *C. higginsianum* and the relationship between them. Moreover, the complete genome has revealed the telomere structure of this fungus and allowed the identification of segmental duplications.

Analysis of the two completely assembled mini-chromosomes showed that both are highly enriched with TEs, which likely explains why they were previously so poorly assembled. The mini-chromosomes of *C. higginsianum* are strikingly different in their content to the 10 larger ‘core’ chromosomes, and they share many of the features that are characteristic of so-called ‘conditionally dispensable’ or ‘accessory’ chromosomes in other plant pathogenic fungi such as *Z. tritici*, *F. fujikuroi, F. oxysporum*, *Alternaria alternata*, *Leptosphaeria maculans* and *Nectria haematococca* [82, 84–88]. Thus, compared to the core chromosomes the mini-chromosomes are repeat-rich and AT-rich, gene-poor, and contain a large proportion of genes of unknown function (up to 75% of the predicted genes). However, in contrast to *F. oxysporum* and *N. haemaococca*, where certain accessory chromosomes are enriched with genes important for pathogenesis [84, 89], gene functional categories related to pathogenicity are almost entirely absent from the mini-chromosomes of *C. higginsianum*. Moreover, fewer genes are transcribed than on the core chromosomes. Nevertheless, we found that the mini-chromosomes are significantly enriched with genes encoding putative effector proteins relative to the core genome, including seven that are highly expressed during infection. Conditionally dispensable chromosomes have been defined as ‘accessory chromosomes that are not required for basic growth but which can confer advantages under certain conditions’ [90]. Functional studies are now required to determine the extent to which the *C. higginsianum* mini-chromosomes carry genes contributing to fungal virulence.

We present here the most comprehensive annotation of repeats available for any *Colletotrichum* species. The overall TE content of *C. higginsianum* (7%) is comparable to that reported previously for *C. graminicola* (12.2%) and *C. orbiculare* (8.3%) [13, 91] and other phytopathogenic fungi with similar sized genomes such as *Z. tritici* (16.7%), *S. sclerotiorum* (12.9%), *B. cinerea* (3.7%) and *V. dahliae* (12.3%) but is strikingly less than in *L. maculans* (33.3%), *Blumeria graminis* f. sp. *hordei* (76.4%) and *Melampsora larici-populina* (51.7%) [6, 8, 25, 92, 93]. LTR elements are the most abundant TEs in *C. higginsianum*, as reported in other fungi, e.g. *Cochliobolus heterostrophus*, *F. fujikuroi*, *F. oxysporum*, *L. maculans* and *V. dahliae* [94–97] and several *Colletotrichum* species [13, 91]. Most of the DNA transposons in *C. higginsianum* belong to the TIR order, superfamily Tc1-Mariner. Thirteen families of these are present as complete copies, suggesting they may be active elements, and they closely resemble Tc1-Mariner elements from *C. incanum*, *C. tofieldiae* and *C. sublineola*. The presence of conserved TEs in species from three sister clades within the *Colletotrichum* phylogeny (namely the Destructivum, Spaethianum and Graminicola clades) [98], but not other members of the genus, suggests they were acquired by a common ancestor. Based on the divergence between TE copies and their consensus sequences, it appears that TIR elements proliferated in the *C. higginsianum* genome relatively recently, whereas most LTR elements are relics of a more ancient burst of transposition. Our finding that some TE families were likely subject to RIP mutation is surprising because RIP occurs during meiosis [78], whereas sexual reproduction has never been reported in *C. higginsianum*. Similarly, RIP-mutated TEs were also detected in another asexual member of the genus, namely *C. cereale* [76]. These authors suggested that RIP may have occurred during an ancestral sexual state or that meiosis occurs cryptically in nature.

An expression analysis of the *C. higginsianum* TEs revealed that 441 copies (~30% of the total TE copies present in the genome) are transcribed in at least one fungal stage, and are therefore likely to be active transposons. Some of these were highly stage-specific in their expression, e.g. in appressoria or during the biotrophic phase. Among those showing the most extreme differential expression, we identified fragments of four LTR retrotransposon families inserted into the 3’UTR regions of five candidate effector proteins expressed in the biotrophic phase. Previously we found fragments of two other retrotransposons, namely CgT1 and Ccret2, in the UTRs of *in planta*-expressed effector genes *ChEC7* and *ChEC10*, respectively [32]. It remains to be determined whether the specific expression patterns of these TEs results from their insertion into the UTRs of stage-specific genes or if *cis*-acting elements contained within their long terminal repeats contribute regulatory information, as reported in some animals and plants [99, 100]. It is interesting to note that in *F. oxysporum* f. sp. *lycopersici*, DNA transposons of the MITE (Miniature Inverted-repeat Transposable Element) family are present in the promoters of many effector genes, including the *SIX* (Secreted In Xylem) genes, but promoter deletion experiments showed that MITEs do not directly regulate the expression of these genes [101].

Analysis of the telomere structure of *C. higginsianum* showed that all 24 subtelomeric regions are characterized by long, highly conserved repeats belonging to one of three families (DTX-chim_G199, DHX-chim_G203, DHX_G198) that share large tracts of homologous sequence. They occur as single, full-length copies showing the same orientation relative to the terminal telomere repeats, and they separate the telomeres from chromosome-unique sequences. All three families contain predicted helicase C and DEAD box domains characteristic of RecQ helicases, which are required for genome maintenance in many organisms [102] and were also identified in the subtelomeres of *Magnaporthe oryzae*, *Z. tritici*, *Saccharomyces cerevisiae* and *Ustilago maydis* [92, 103–105]. However, other motifs characteristic of RecQ helicases, namely zinc-binding and RQC DNA-binding domains, were not detectable. DTX-chim_G199, which is located at seven subtelomeres in *C. higginsianum*, additionally contains HTH and DDE motifs, both typical of transposases, and it probably arose from DHX-G198 by the insertion of a DNA-TIR transposon (Fig. 5). On the other hand, DHX-chim-G203, present at four subtelomeres, was derived from DHX-G198 by the insertion of a chromodomain-containing LTR-Gypsy retrotransposon. LTR-Gypsy elements were likewise detected in the subtelomeres of *F. graminearum* [83], while non-LTR retrotransposons were found to be associated with telomeres in *Z. tritici* [92] and perennial rye grass-infecting isolates of *M. oryzae*, where they were termed ‘MoTeRs’ [106]. MoTeRs promote extreme telomere variability in *M. oryzae*, but unlike the retrotransposon present in DHX-chim-G203, they insert exclusively into the terminal telomere repeat tract.

A genome-wide survey revealed the presence of six segmental duplications (SDs) in *C. higginsianum*, two of which involved the duplication of putative pathogenicity factors that are transcribed during infection. Notably, four SDs are situated near chromosome ends and are bordered by the conserved subtelomeric repeats. The presence of these large, highly similar repeats at *C. higginsianum* subtelomeres may predispose the adjacent genomic regions to undergo segmental duplication through non-allelic homologous recombination. The intra-chromosomal SD6 may also have resulted from homology-based recombination involving an interstitial fragment of DHX-chim-G203, causing a sequence inversion. However, two SDs were neither located near telomeres nor associated with flanking repetitive sequences, suggesting they may instead have arisen through the repair of double-strand DNA breaks by non-homologous end-joining. Recent work on *V. dahliae*, a phytopathogenic fungus that is phylogenetically close to *Colletotrichum*, has highlighted the importance of SDs in generating hyper-variable, lineage-specific genomic regions that are enriched with virulence-related genes [107]. These authors also proposed that the duplication of genomic regions *via* mitotic crossing-over provides an important source of genetic diversity in asexual pathogens such as *V. dahliae* that do not undergo meiotic recombination [108]. Likewise, sexual reproduction was never observed in *C. higginsianum* [13]. Thus, segmental duplication mediated by recombination between the subtelomeric repeats could provide a mechanism to amplify and diversify genes, and thereby accelerate host adaptation, in this asexual pathogen.

In the previous *C. higginsianum* gene annotation, the number of predicted SMKGs (103) was over-estimated, while the number of SM gene clusters (47) was under-estimated due to their fragmentation between contigs [13]. The more accurate annotation presented here confirms that *C. higginsianum* encodes one of the largest repertoires of SMKGs (89) and SM gene clusters (77) of any sequenced ascomycete [88, 109–112], suggesting a large capacity to produce diverse metabolites. Interestingly, we detected a statistically significant association of SM cluster genes with 10 families of retrotransposons and DNA transposons. Previously, we also found that 71% of SM clusters in *C. graminicola* co-localize with TEs [13], and TEs are similarly enriched in regions flanking the secondary metabolism genes of *Epichloe festucae* and several Dothidiomycete species [113, 114]. Proximity to TEs potentially exposes genes to higher rates of repeat-induced point mutation, and therefore accelerated evolution [114, 115]. Moreover, TE copies belonging to the same or highly similar families provide sites for ectopic recombination [116], which may result in deletions [113] or the creation of new clusters with new combinations of genes, thereby increasing chemotypic diversity [117].

A striking feature of secondary metabolism in *C. higginsianum* is that the majority (60%) of expressed SM gene clusters are only transcribed during plant infection, notably in penetrating appressoria and/or the biotrophic phase, and not in vitro. The putative products of these infection-specific SM clusters are unknown and phylogenetic analyses identified only two clusters in which the key gene belongs to a clade with a characterized end-product, namely ChPKS26 (3-methylorcinaldehyde) and ChPKS12 (cercosporin). The plant-derived signal(s) that presumably trigger the co-ordinated expression of these clusters, and the mechanisms underlying their tight regulation, remain unknown. The pChPKS38::RFP reporter strain described here will provide a ‘biosensor’ to search for such plant signals and to identify fungal genes required for their perception and transduction. In *Aspergillus fumigatus*, mammalian infection is associated with the co-ordinated expression of SM gene clusters located near telomeres [118]. However, in *C. higginsianum*, although 15 clusters are located less than 200 kb from a telomere, only 3 of these are induced at any stage of plant infection. Accumulating evidence points to the critical role of chromatin status in regulating the expression of SM gene clusters in filamentous fungi [119–122]. The genome-wide analysis of post-translational histone modifications such as methylation and acetylation, as well as DNA base modifications, will be greatly facilitated by the availability of a high-quality genome assembly for *C. higginsianum*.

## Conclusions

Our study demonstrates that access to a complete genome assembly is invaluable for the analysis of genomic features such as transposable elements, telomeres, structural rearrangements and large gene clusters. We show that the mini-chromosomes of *C. higginsianum* differ markedly from the core genome in their gene and repeat content and resemble the conditionally dispensable chromosomes of some other plant pathogenic fungi. Analysis of the TE landscape in *C. higginsianum* provided new insights into the potential role of TEs in gene and genome evolution in this fungus. Thus, repeat-mediated segmental duplication was identified as a possible mechanism for generating genetic diversity in this fungus. Moreover, the co-localization of particular families of retrotransposons and DNA transposons with SM gene clusters and effector genes raises the possibility that TEs accelerate the evolution of these pathogenicity-related genes, for example by introducing mutations or generating new gene combinations through ectopic recombination. The comprehensive inventory of SM gene clusters described here reveals a large potential for discovering novel bioactive molecules from *C. higginsianum* and will expedite identification of the corresponding biosynthetic pathways. Finally, the high-quality genome assembly provides a reference for comparison with additional *C. higginsianum* isolates and other members of the genus, and will facilitate future functional genomics in this important model pathogen.

## Additional files

Additional file 1: Primers used in this study. (PDF 40 kb)

Additional file 2: Genbank accession numbers of *Colletotrichum* transposon sequences used in the REPET annotation pipeline. (PDF 81 kb)

Additional file 3: List of all PKS and PKS-NRPS protein sequences used in the phylogenetic analysis. (XLSX 19 kb)

Additional file 4: Summary of unitigs comprising the *C. higginsianum* assembly. (PDF 91 kb)

Additional file 5: **(A)** PCR products encompassing the two break-points shown in Fig. 1 confirm the unitig sequences rather than the optical maps. PCR validation of putative break-points using primer pairs P812 - P813 and P422 - P423. If the assembly is correct, the expected PCR products are respectively 3992 bp and 5470 bp long. L: Generuler 1 kb Plus DNA Ladder; 1: unitig_7 break-point; 2: unitig_9 break-point. **(B)** PCR validation of segmental duplication SD2. Primers P677, P678 and P679 are colour-coded for the features they match. L: Generuler 1 kb Plus DNA Ladder; 3: P678xP677 amplicon; 4: P679xP677 amplicon. (PDF 2434 kb)

Additional file 6: Characteristics and contents of the 12 largest unitigs in the new genome assembly, corresponding to the 12 chromosomes of *C. higginsianum*. (PDF 129 kb)

Additional file 7: Correspondence between old and new gene IDs. (XLSX 359 kb)

Additional file 8: **(A)** Gene category predictions and transcriptomic analysis by RNA-Seq of four developmental stages of *C. higginsianum*. VA: appressoria in vitro, PA: appressoria *in planta*, BP: biotrophic phase, NP: necrotrophic phase. **(B)** Annotation of secondary metabolism key genes and clusters. (XLSX 4943 kb)

Additional file 9: List of the secondary metabolism key genes and their catalytic domains. (PDF 132 kb)

Additional file 10: Schematic representation of the 77 secondary metabolism gene clusters of *C. higginsianum*. (PDF 374 kb)

Additional file 11: Phylogenetic analysis of the 40 PKS and 6 PKS-NRPS hybrids of *C. higginsianum*. KS and AT domains were aligned with 230 enzymes from other fungi (Additional file 3). *C. higginsianum* genes are represented with a green font. Red diamonds represent SMKGs induced specifically in at least one of the three stages of plant infection investigated. Where a *C. higginsianum* protein belongs to a clade containing a well-characterized protein linked to a metabolite, the structure of that metabolite is shown. The PR-PKS clade is represented only in the complete version of the tree. *Caenorhabditis elegans*, *Gallus gallus* and *Homo sapiens* FAS are used as outgroups. (PDF 2154 kb)

Additional file 12: Comparison of gene content between characterized secondary metabolism gene clusters and orthologous *C. higginsianum* clusters (.xlsx) (XLSX 18 kb)

Additional file 13: Size distribution of Simple Sequence Repeats (SSR) in the *C. higginsianum* genome. (PDF 245 kb)

Additional file 14: Genome content and characteristics of 41 transposable element families in *C. higginsianum*. (PDF 521 kb)

Additional file 15: Phylogenetic analysis of 43 cytosine-specific methyltransferase domains (PF00145) from Dnmt1 fungal proteins and *S. Pombe* DNMT2, which was used as an outgroup. Analysis was performed as described previously [25]. Only clades with bootstrap support greater than 70% are represented. Red: *C. higginsianum* proteins; Blue: Basidiomycetes. (PDF 3801 kb)

Additional file 16: **(A)** Dinucleotide mutation bias among TE copies belonging to different TE orders. Mutation rates were calculated using RIPCAL by comparing each TE copy with a Ti/Tv > 1.5 to the TE consensus sequence. Y-axis: percentage relative to the total number of copies used in RIPCAL analysis. Coloured bars indicate the percentage of copies with expected RIP (Ti/Tv > 1.5) and dinucleotide preferentially used (>1/3) in CN- > TN and (cNG - > cNA) mutations. Black bar: percentage of copies without expected RIP (Ti/Tv > 1.5). Gray bar: percentage of copies with expected RIP (Ti/Tv > 1.5) but no evidence of dinucleotide bias. **(B)** Plot showing the sequence divergence of TE copies belonging to different TE orders relative to their respective consensus sequences. (PDF 466 kb)

Additional file 17: Plot showing the distribution of *C. higginsianum* transposable element families across the 25 largest unitigs. (PDF 193 kb)

Additional file 18: Schematic representation of the distribution of three families of conserved repeats at the 24 subtelomeres of *C. higginsianum*. (PDF 1186 kb)

Additional file 19: Expression analysis of *C. higginsianum* transposable elements (TEs). **(A)** Distribution of log (CPM) per condition with multi-mapped read counts. **(B)** Distribution of log (CPM) per condition with uniquely mapped read counts. (PDF 1853 kb)

Additional file 20: Expression of TE copies based on average log (CPM), with a threshold of 1.35. Results are displayed for uniquely-mapped and multi-mapped read counts. (PDF 299 kb)

Additional file 21: Stage-specific expression of *C. higginsianum* transposable elements (TEs). **(A)** Heatmap showing the expression profiles of TEs. **(B)** K-means clustering of 441 TE copies considered to be expressed in at least one fungal stage (VA = in vitro appressoria, PA = *in planta* appressoria, BP = biotrophic phase). For each of the five clusters, the average profile is shown in red. **(C)** Localization of *in planta*-expressed LTR transposon fragments in the 3’ UTR regions of *C. higginsianum* effector genes. IGV screenshots showing the genomic locations of TE copies RXX_R113 and RLX_P25.13 (red) in relation to effector genes ChEC35 and ChEC117 (green), respectively. RNA-Seq reads are displayed for appressoria in vitro (VA) and the biotrophic phase (BP). The RLX_P25.13 copy comprises a ‘solo’-LTR, likely produced by homologous recombination between two LTRs, leading to deletion of the internal retrotransposon sequence. (PDF 2850 kb)

Additional file 22: Genomic locations of TE copies from clusters 2 and 3 showing extreme expression profiles. (PDF 222 kb)

Additional file 23: Results of permutation tests for the association of transposon families with secondary metabolism and effector genes. (PDF 363 kb)

Additional file 24: Characteristics of six segmental duplications identified in the *C. higginsianum* genome assembly. (PDF 257 kb)

Additional file 25: Gene content of six segmental duplications identified in the *C. higginsianum* genome assembly and polymorphisms between the duplicated genes. (PDF 319 kb)

Additional file 26: Schematic representation of the association between four segmental duplications (shown in green) with the *C. higginsianum* subtelomeric repeats DHX_G198, DTX_chim-G199, and DHX_chim-G203. Homologous regions are shaded grey-blue. (PDF 747 kb)

## Abbreviations

AT: acyl transferase
BP: biotrophic phase
CPM: counts per million mapped reads
CSEP: candidate secreted effector protein
DMATS: dimethylallyltryptophan synthase
FC: fold change
FLC: full-length copy
HGAP: hierarchical genome assembly process
HR-PKS: highly-reducing PKS
KS: ketoacyl synthase
LTR: long terminal repeat
NP: necrotrophic phase
NR-PKS: non-reducing PKS
NRPS: non-ribosomal peptide synthetase
ORF: open reading frame
PA: appressoria *in planta*
PCR: polymerase chain reaction
PDA: potato dextrose agar
PKS: polyketide synthase
PKS-NRPS: hybrid PKS-NRPS
RAL: resorcylic acid lactone
RIP: repeat-induced point mutation
SD: segmental duplication
SGS: second-generation sequencing
SM: secondary metabolism
SMKG: secondary metabolism key enzyme-encoding gene
SMRT: single-molecule real-time
SNP: single nucleotide polymorphism
SSR: simple sequence repeats
TE: transposable element
TGS: third-generation sequencing
TIR: terminal inverted repeat
TPM: transcripts per million
TS: terpene synthase
UTR: untranslated region
VA: appressoria in vitro
