# Supplementary material for: Gapless genome assembly of Colletotrichum higginsianum reveals chromosome structure and association of transposable elements with secondary metabolite gene clusters
Source: BMC Genomics. 2017 Aug 29;18:667. doi: 10.1186/s12864-017-4083-x (PMC5576322; doi:10.1186/s12864-017-4083-x)
Supplement: Supplementary file 1 — Primers used in this study. (PDF 40 kb) [file 12864_2017_4083_MOESM1_ESM.pdf]

Additional File 1: Primers used in this study.

Designation	Sequence 5' to 3'	Notes
Diag677	GGGGTGCCTTGTCTCTTGAAGC	Validation of the segmental duplication between unitigs 8 and 9
Diag678	CAGATATCGCTCATTGGACACGA	
Diag679	GAGAAGATTGACGTTTGTACCTCGT	
Diag422	CAGGTATCCTCGACAGTCTCACT	Validation of the chromosomal translocation
Diag423	GTAGAGCTCAGCACGGATGAC	
Diag812	CTACCCCGTCATCGTATCGCC	Validation of the chromosomal translocation
Diag813	AGGGAGGGAGGGACCCTACAC	
Diag432	ACGAGGATTCTCCACGAAACA	Validation of contigs joining of the chromosome 11
Diag433	GTATGCGCATCATTATCCAACTC	
P1-F	TGTCTTTCCCGGCATGATAG	5' flanking region of *ChPKS38*
P1-R	ctcggaggaggccatTTTCGCAATAGTGAGCGTGT	
P2-F	gacgagttcttctgaATCTCATTGAAGCCGCTGTG	3' flanking region of *ChPKS38*
P2-R	CGTCGACCAAGACGAGCA	
P3-F	ctcactattgcgaaaATGGCCTCCTCCGAGGA	RFP fragment
P3-R	cactgtacagagctcTGCTGCAAGGCGATTAAGT	
P4-F	aatcgccttgcagcaGAGCTCTGTACAGTGACCGGT	Marker of selection
P4-R	cggcttcaatgagatTCAGAAGAACTCGTCAAGAAGG	
P5-F	TGACTGCGCACTTCCACAG	Nested primers
P5-R	GCAAATCGCTGGATCTGAGAC	
