# Supplementary material for: Gapless genome assembly of Colletotrichum higginsianum reveals chromosome structure and association of transposable elements with secondary metabolite gene clusters
Source: BMC Genomics. 2017 Aug 29;18:667. doi: 10.1186/s12864-017-4083-x (PMC5576322; doi:10.1186/s12864-017-4083-x)
Supplement: Supplementary file 2 — Genbank accession numbers of Colletotrichum transposon sequences used in the REPET annotation pipeline. (PDF 81 kb) [file 12864_2017_4083_MOESM2_ESM.pdf]

Additional file 2: Genbank accession numbers of *Colletotrichum* transposon sequences used in the REPET annotation pipeline

Accession No.	Species/isolate	Description	Sequence	Length (bp)
JX121209.1	*Glomerella graminicola*	TCg1	complete	1866
JF313218.1	*Glomerella lindemuthiana*	RETROCL1	complete	634
L76205.1	*Colletotrichum gloeosporioides*	COGNLRC CgT1		5812
AF264028.1	*Colletotrichum gloeosporioides*	Cgret retrotransposon		6999
DQ663513.1	*Colletotrichum cereale* isolate PA-50231 clone A15	Ccret2 retrotransposon	partial	1690
DQ663512.1	*Colletotrichum cereale* isolate PA-50005 clone	Ccret2 POL3gag11 retrotransposon	partial	4478
DQ663511.1	*Colletotrichum cereale* isolate PA-50005 clone DBP16	Ccret2 retrotransposon	partial	2380
DQ663510.1	*Colletotrichum cereale* isolate PA-50005 clone DBP6	Ccret1 retrotransposon	partial	1313
EF067893.1	*Colletotrichum cereale* clone 9F8-1787	Ccret1 LTR retrotransposon	partial	1784
EF067894.1	*Colletotrichum cereale* clone 9F8-2137	Ccret3 non-LTR retrotransposon	partial	2137
EF067892.1	*Colletotrichum cereale* clone 9F8-1558	Ccret3 non-LTR retrotransposon	partial	1556
