# Supplementary material for: Gapless genome assembly of Colletotrichum higginsianum reveals chromosome structure and association of transposable elements with secondary metabolite gene clusters
Source: BMC Genomics. 2017 Aug 29;18:667. doi: 10.1186/s12864-017-4083-x (PMC5576322; doi:10.1186/s12864-017-4083-x)
Supplement: Supplementary file 4 — Summary of unitigs comprising the C. higginsianum assembly. (PDF 91 kb) [file 12864_2017_4083_MOESM4_ESM.pdf]

Additional File 4: Summary of unitigs comprising the *Colletotrichum higginsianum* genome assembly

Chromosome no.	Unitig ID	NCBI accession no.	Sequence length (bp)	5'-telomere	3'-telomere	Comment
1	unitig_1	NC_030954.1	6,042,495	✓	✓	
2	unitig_2	NC_030955.1	6,014,962	✓	✓	
3	unitig_3	NC_030956.1	5,992,392	✓	✓	
4	unitig_4	NC_030957.1	5,325,643	✓	✓	
5	unitig_5	NC_030958.1	5,201,688	✓	✓	
6	unitig_6	NC_030959.1	4,523,820	✓	✓	
7	unitig_7	NW_017263917.1	4,439,061		✓	missing 5' telomere and rDNA repeats
8	unitig_8	NC_030960.1	4,422,509	✓	✓	
9	unitig_9	NC_030961.1	4,092,876	✓	✓	
10	unitig_10	NC_030962.1	3,084,912	✓	✓	
11	unitig_11	NC_030963.1	646,208	subtelomere	✓	5' subtelomeric repeats only
12	unitig_12	NC_030964.1	597,935	✓	✓	
7	unitig_13	NW_017263918.1	34,771	✓		rDNA repeats and 5' telomere
7	unitig_14	NW_017263919.1	29,996			rDNA repeats
7	unitig_15	NW_017263920.1	28,150			rDNA repeats
7	unitig_16	NW_017263921.1	27,823			rDNA repeats
7	unitig_17	NW_017263922.1	27,135			rDNA repeats
7	unitig_18	NW_017263923.1	27,003			rDNA repeats
7	unitig_19	NW_017263924.1	26,176			rDNA repeats
7	unitig_20	NW_017263925.1	25,803			rDNA repeats
7	unitig_21	NW_017263926.1	23,831			rDNA repeats
7	unitig_22	NW_017263927.1	22,425			rDNA repeats
7	unitig_23	NW_017263928.1	21,781			rDNA repeats
7	unitig_24	NW_017263929.1	19,697			rDNA repeats
7	unitig_25	NW_017263930.1	17,011			rDNA repeats
Mitochondrial genome	unitig_26	Not submitted	68,054			
Mitochondrial genome	unitig_27	Not submitted	17,885			
Mitochondrial genome	unitig_28	Not submitted	17,219			
