# Supplementary figures and images for: Gapless genome assembly of Colletotrichum higginsianum reveals chromosome structure and association of transposable elements with secondary metabolite gene clusters
Source: BMC Genomics. 2017 Aug 29;18:667. doi: 10.1186/s12864-017-4083-x (PMC5576322; doi:10.1186/s12864-017-4083-x)

**A**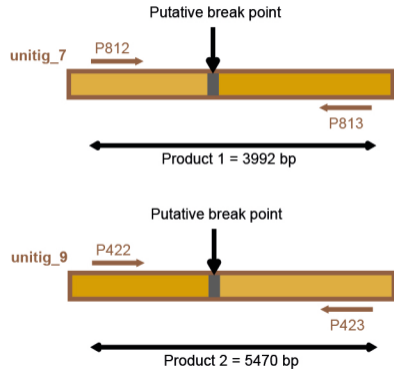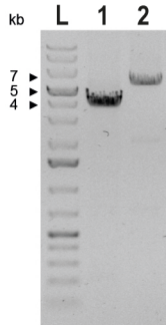**B**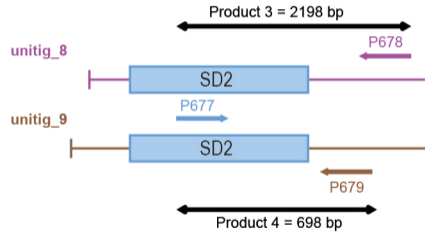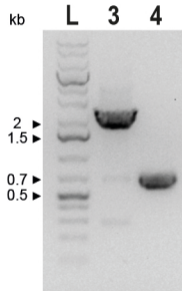

Supplement: Supplementary file 5 — (A) PCR products encompassing the two break-points shown in Fig. 1 confirm the unitig sequences rather than the optical maps. PCR validation of putative break-points using primer pairs P812 - P813 and P422 - P423. If the assembly is correct, the expected PCR products are respectively 3992 bp and 5470 bp long. L: Generuler 1 kb Plus DNA Ladder; 1: unitig_7 break-point; 2: unitig_9 break-point. (B) PCR validation of segmental duplication SD2. Primers P677, P678 and P679 are colour-coded for the features they match. L: Generuler 1 kb Plus DNA Ladder; 3: P678xP677 amplicon; 4: P679xP677 amplicon. (PDF 2434 kb) [file 12864_2017_4083_MOESM5_ESM.pdf]

# Additional File 10

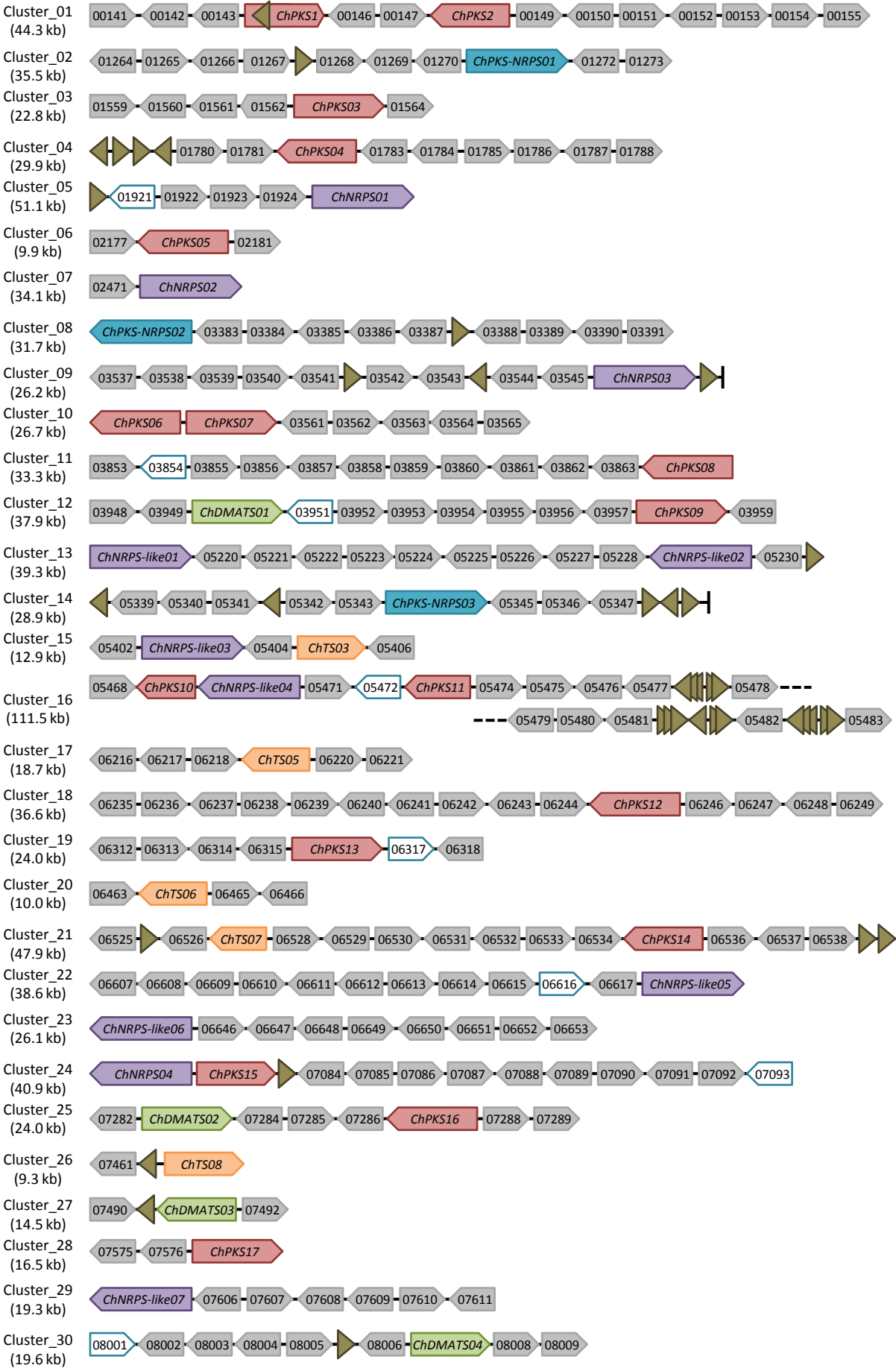

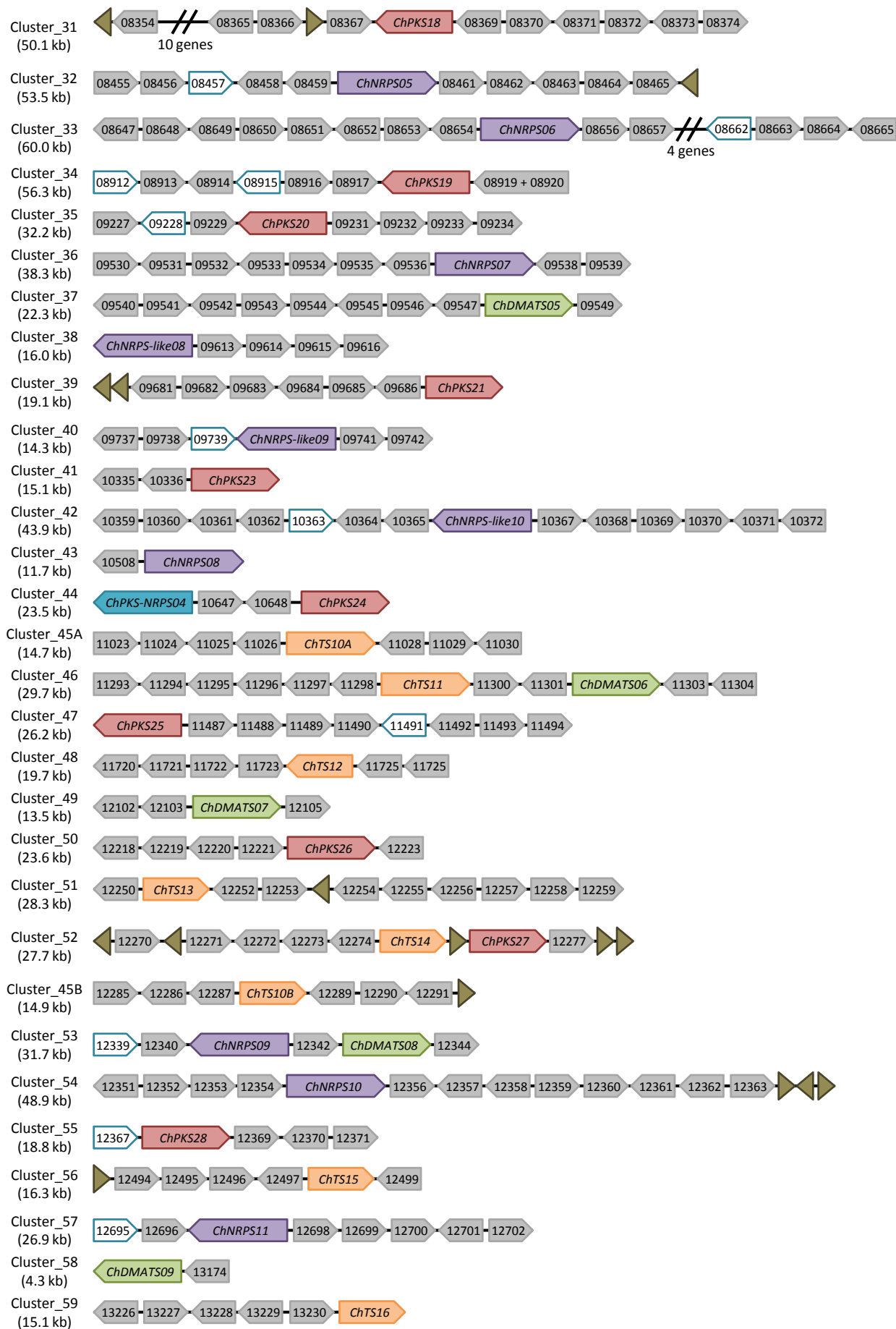

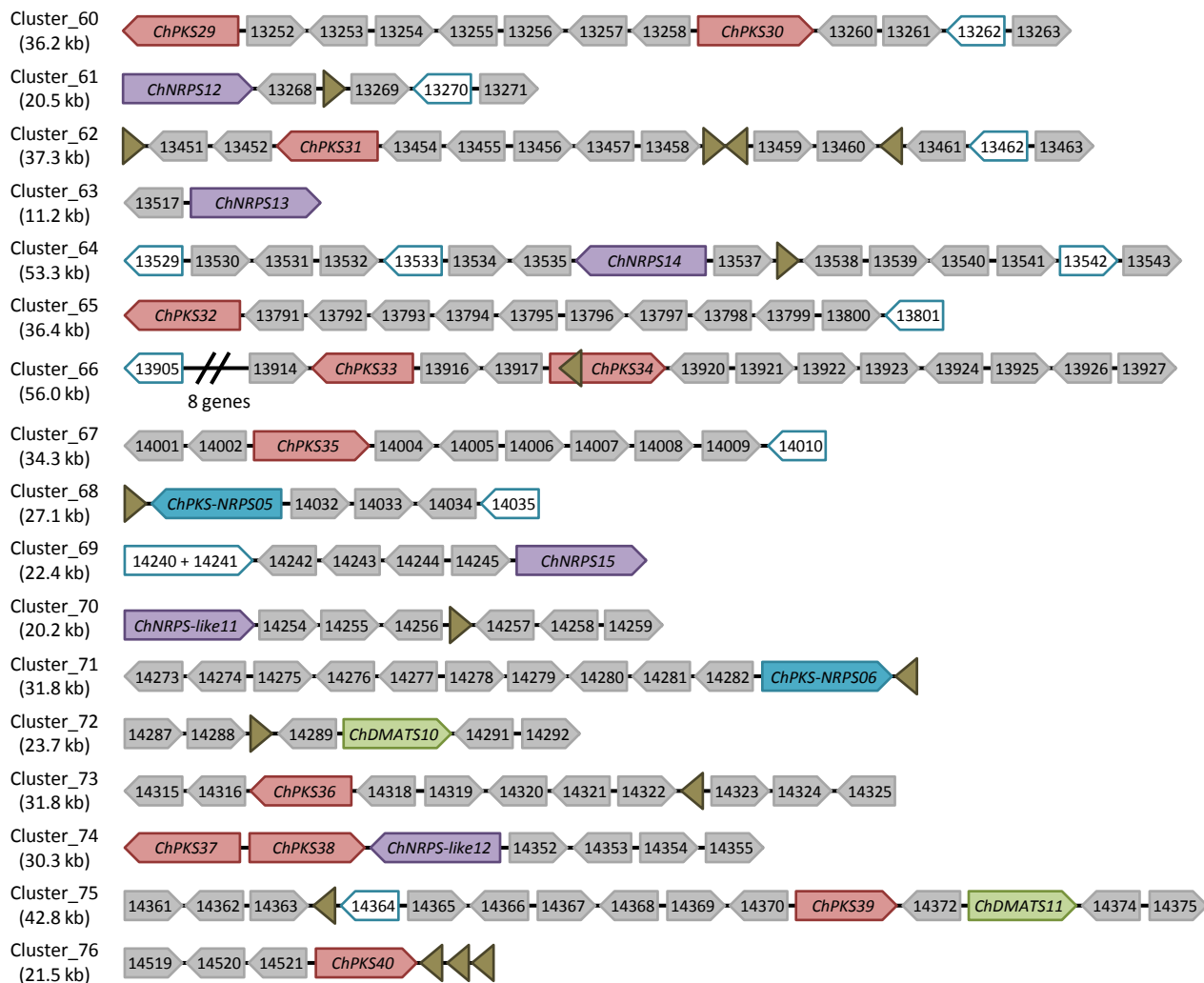

## Legend:

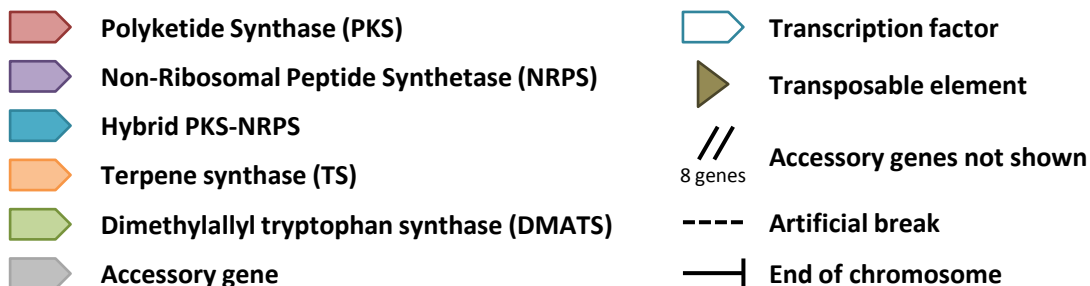

Supplement: Supplementary file 10 — Schematic representation of the 77 secondary metabolism gene clusters of C. higginsianum. (PDF 374 kb) [file 12864_2017_4083_MOESM10_ESM.pdf]

# Additional File 11

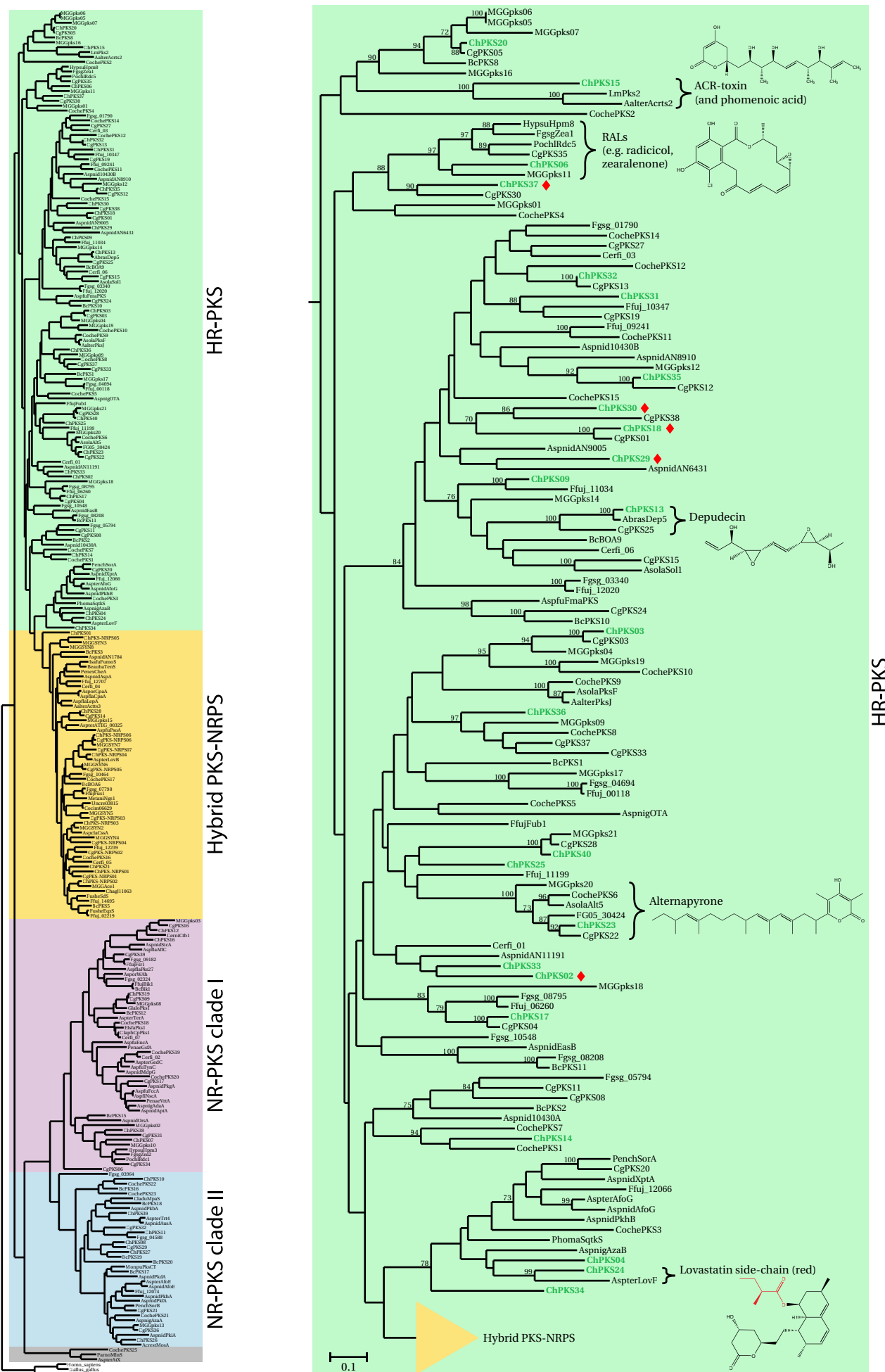

(continued, next page)

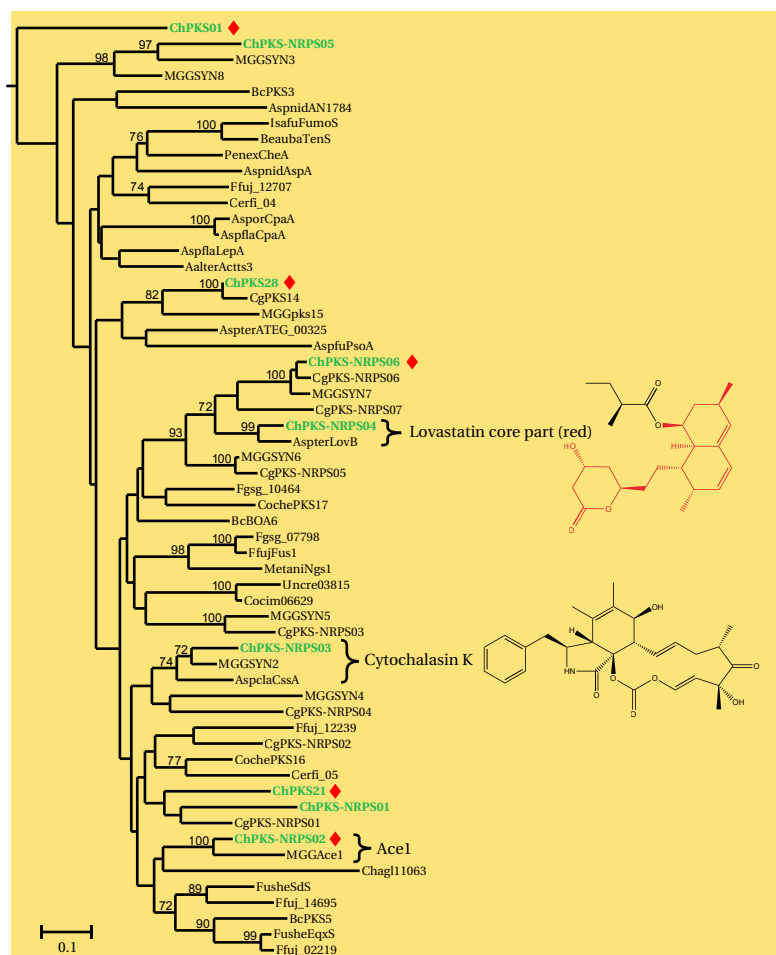

Hybrid PKS-NRPS

(continued, next page)

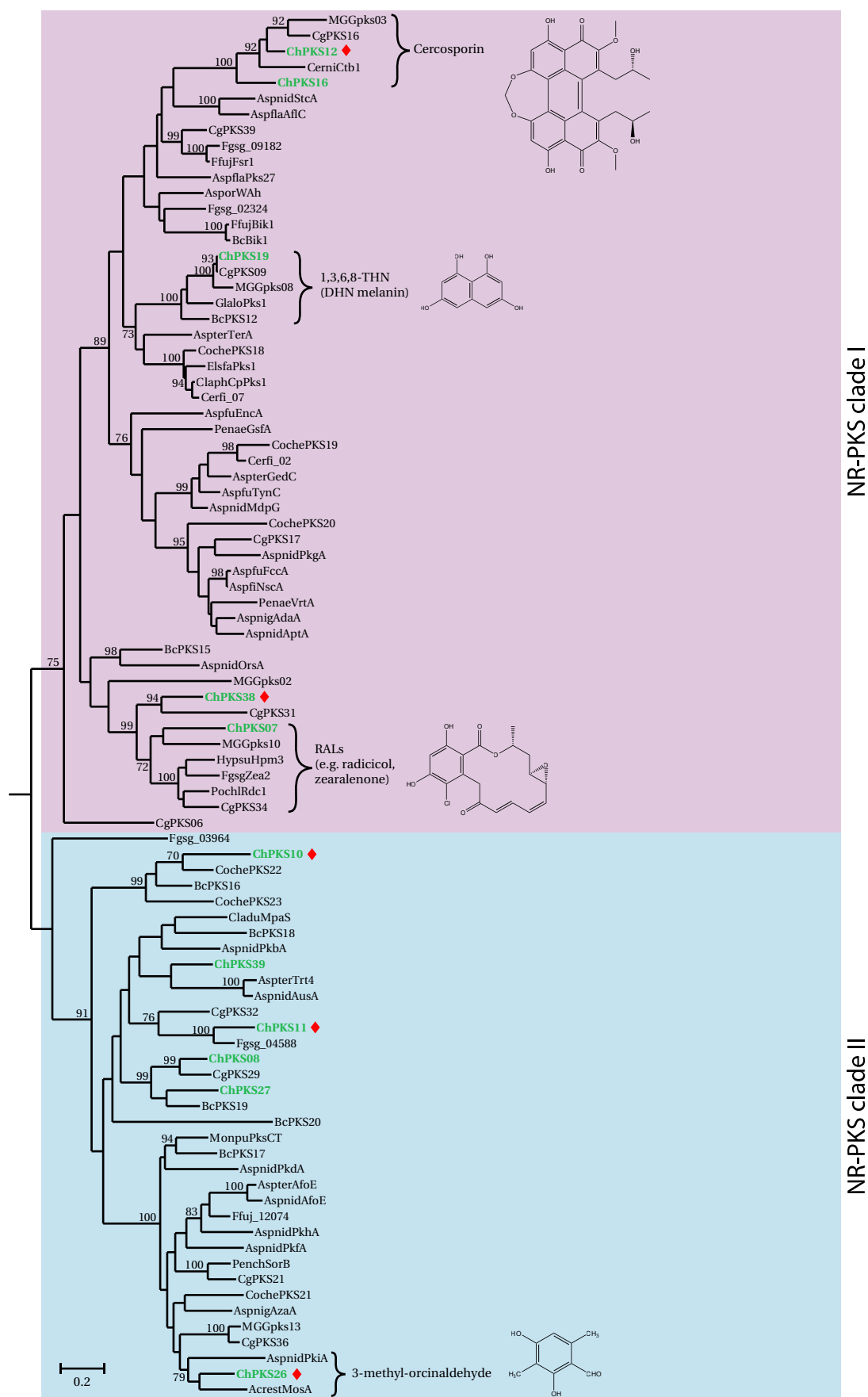

Supplement: Supplementary file 11 — Phylogenetic analysis of the 40 PKS and 6 PKS-NRPS hybrids of C. higginsianum. KS and AT domains were aligned with 230 enzymes from other fungi (Additional file 3). C. higginsianum genes are represented with a green font. Red diamonds represent SMKGs induced specifically in at least one of the three stages of plant infection investigated. Where a C. higginsianum protein belongs to a clade containing a well-characterized protein linked to a metabolite, the structure of that metabolite is shown. The PR-PKS clade is represented only in the complete version of the tree. Caenorhabditis elegans, Gallus gallus and Homo sapiens FAS are used as outgroups. (PDF 2154 kb) [file 12864_2017_4083_MOESM11_ESM.pdf]

Additional File 15

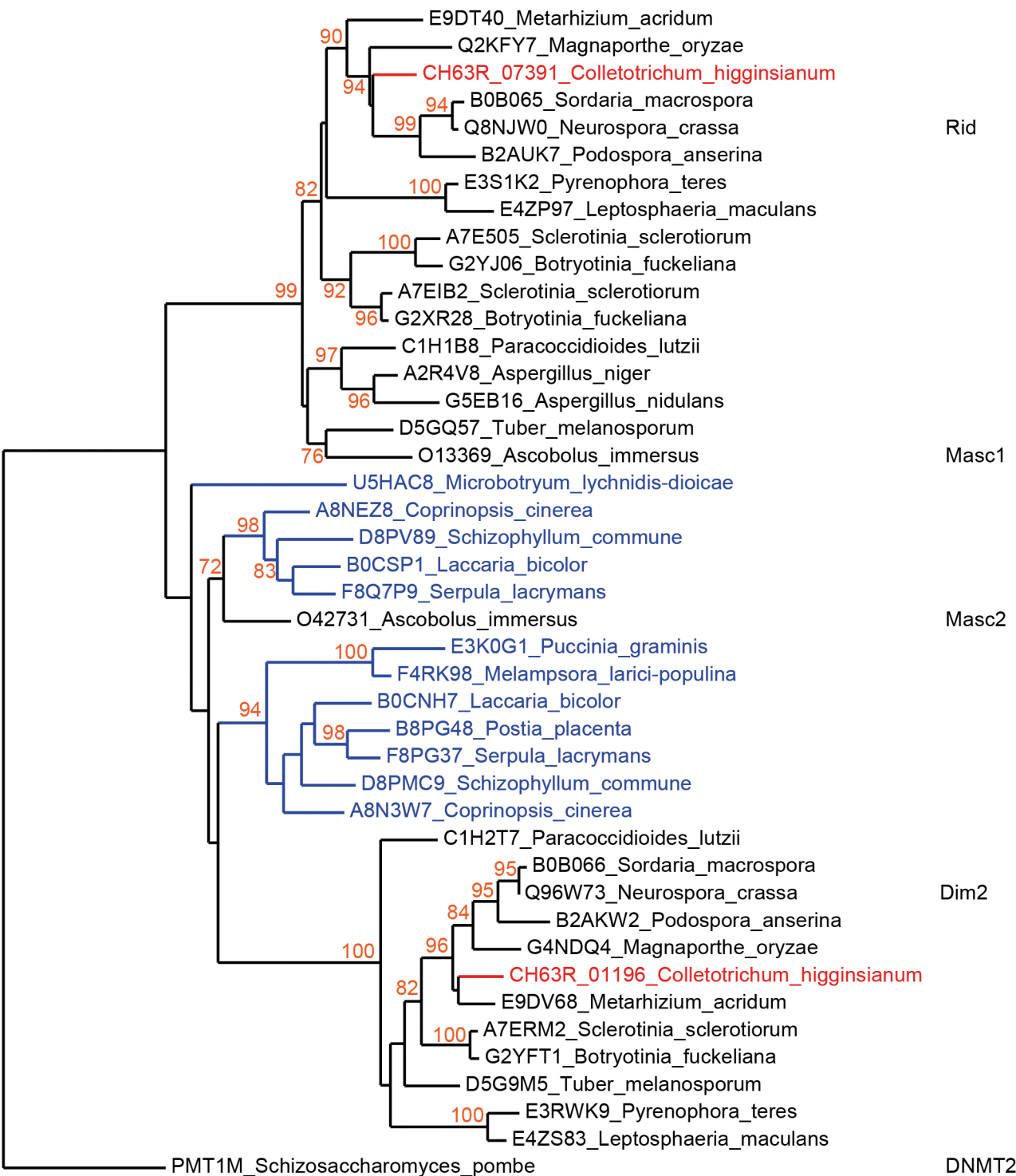

Supplement: Supplementary file 15 — Phylogenetic analysis of 43 cytosine-specific methyltransferase domains (PF00145) from Dnmt1 fungal proteins and S. Pombe DNMT2, which was used as an outgroup. Analysis was performed as described previously [25]. Only clades with bootstrap support greater than 70% are represented. Red: C. higginsianum proteins; Blue: Basidiomycetes. (PDF 3801 kb) [file 12864_2017_4083_MOESM15_ESM.pdf]

# Additional file 16

A

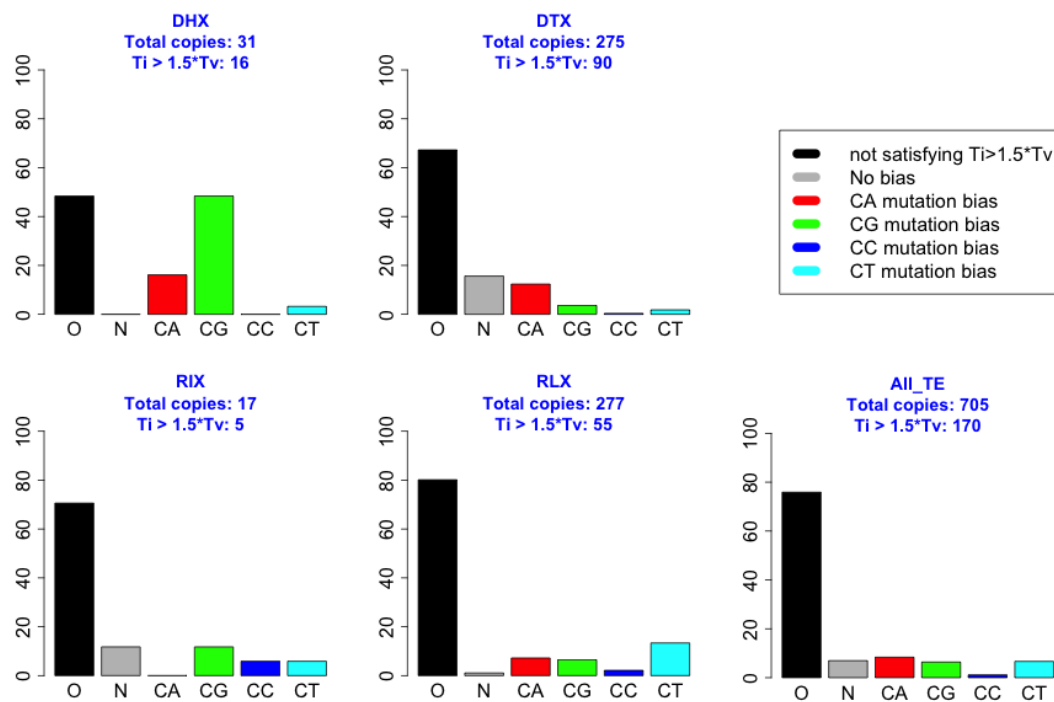

B

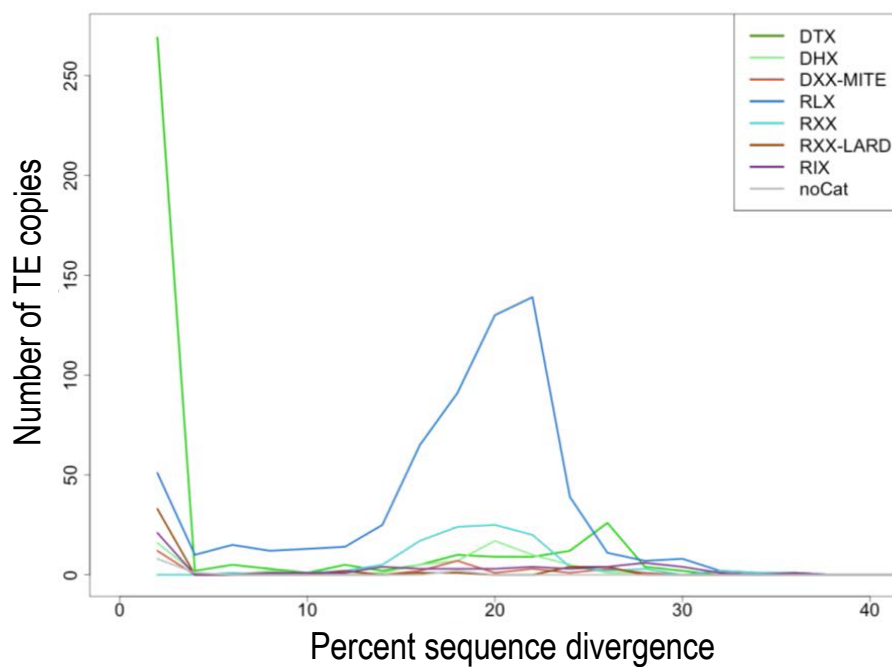

Supplement: Supplementary file 16 — (A) Dinucleotide mutation bias among TE copies belonging to different TE orders. Mutation rates were calculated using RIPCAL by comparing each TE copy with a Ti/Tv > 1.5 to the TE consensus sequence. Y-axis: percentage relative to the total number of copies used in RIPCAL analysis. Coloured bars indicate the percentage of copies with expected RIP (Ti/Tv > 1.5) and dinucleotide preferentially used (>1/3) in CN- > TN and (cNG - > cNA) mutations. Black bar: percentage of copies without expected RIP (Ti/Tv > 1.5). Gray bar: percentage of copies with expected RIP (Ti/Tv > 1.5) but no evidence of dinucleotide bias. (B) Plot showing the sequence divergence of TE copies belonging to different TE orders relative to their respective consensus sequences. (PDF 466 kb) [file 12864_2017_4083_MOESM16_ESM.pdf]

# Additional File 19

A

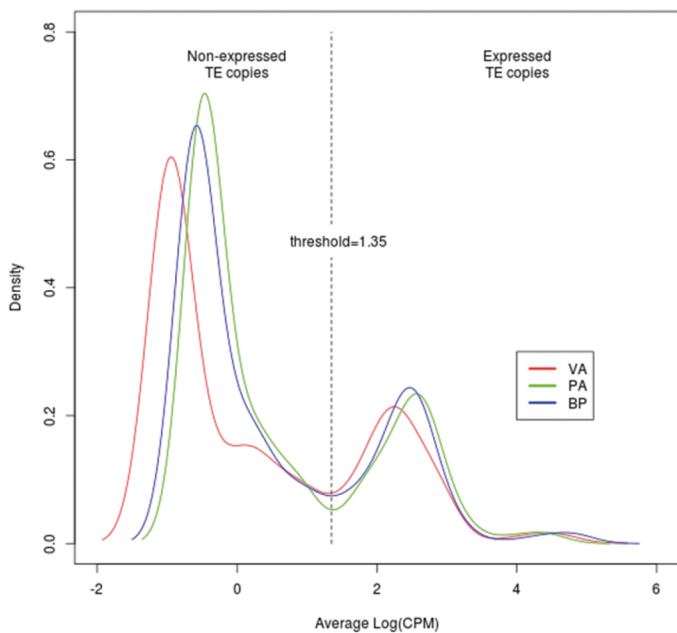

B

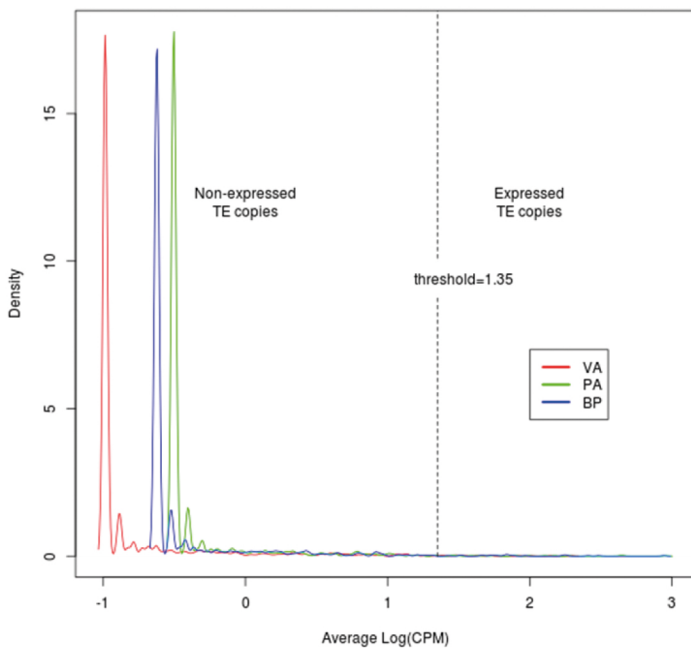

Supplement: Supplementary file 19 — Expression analysis of C. higginsianum transposable elements (TEs). (A) Distribution of log (CPM) per condition with multi-mapped read counts. (B) Distribution of log (CPM) per condition with uniquely mapped read counts. (PDF 1853 kb) [file 12864_2017_4083_MOESM19_ESM.pdf]

# Additional File 21

A

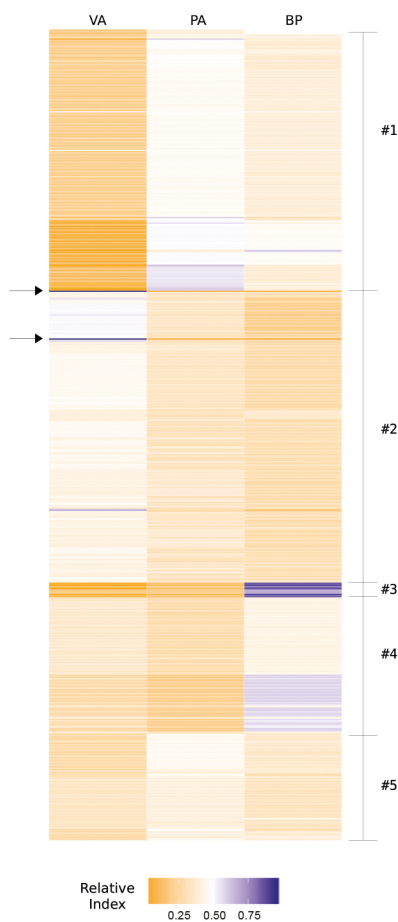

B

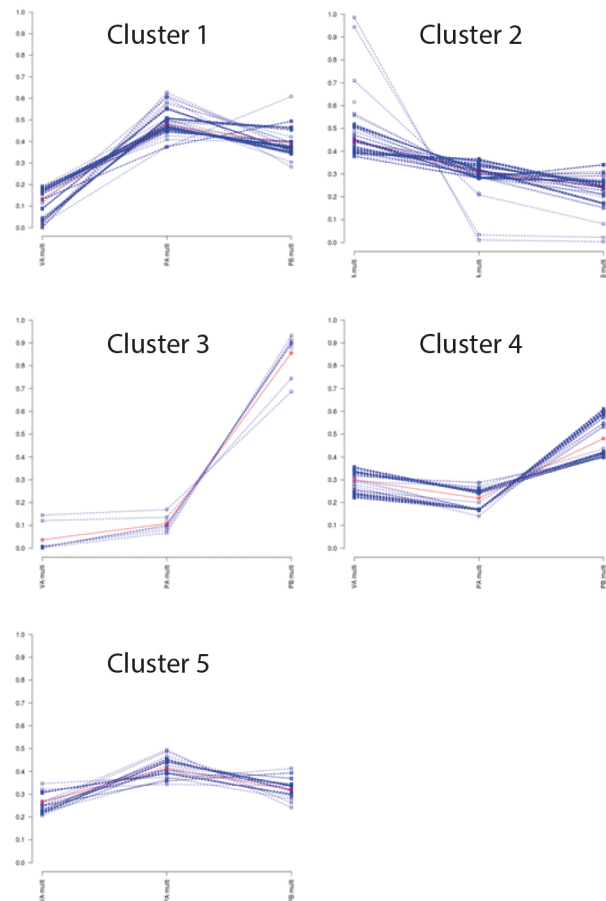

C

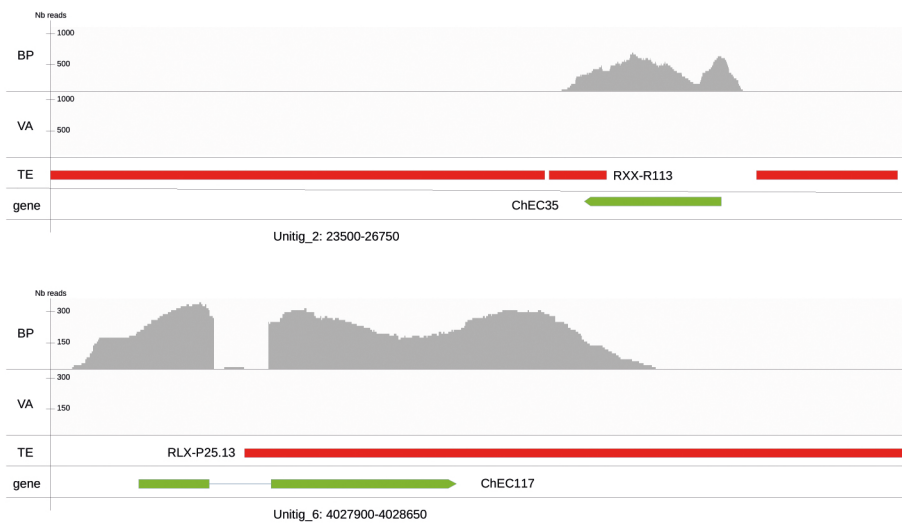

Supplement: Supplementary file 21 — Stage-specific expression of C. higginsianum transposable elements (TEs). (A) Heatmap showing the expression profiles of TEs. (B) K-means clustering of 441 TE copies considered to be expressed in at least one fungal stage (VA = in vitro appressoria, PA = in planta appressoria, BP = biotrophic phase). For each of the five clusters, the average profile is shown in red. (C) Localization of in planta-expressed LTR transposon fragments in the 3’ UTR regions of C. higginsianum effector genes. IGV screenshots showing the genomic locations of TE copies RXX_R113 and RLX_P25.13 (red) in relation to effector genes ChEC35 and ChEC117 (green), respectively. RNA-Seq reads are displayed for appressoria in vitro (VA) and the biotrophic phase (BP). The RLX_P25.13 copy comprises a ‘solo’-LTR, likely produced by homologous recombination between two LTRs, leading to deletion of the internal retrotransposon sequence. (PDF 2850 kb) [file 12864_2017_4083_MOESM21_ESM.pdf]

Additional file 26

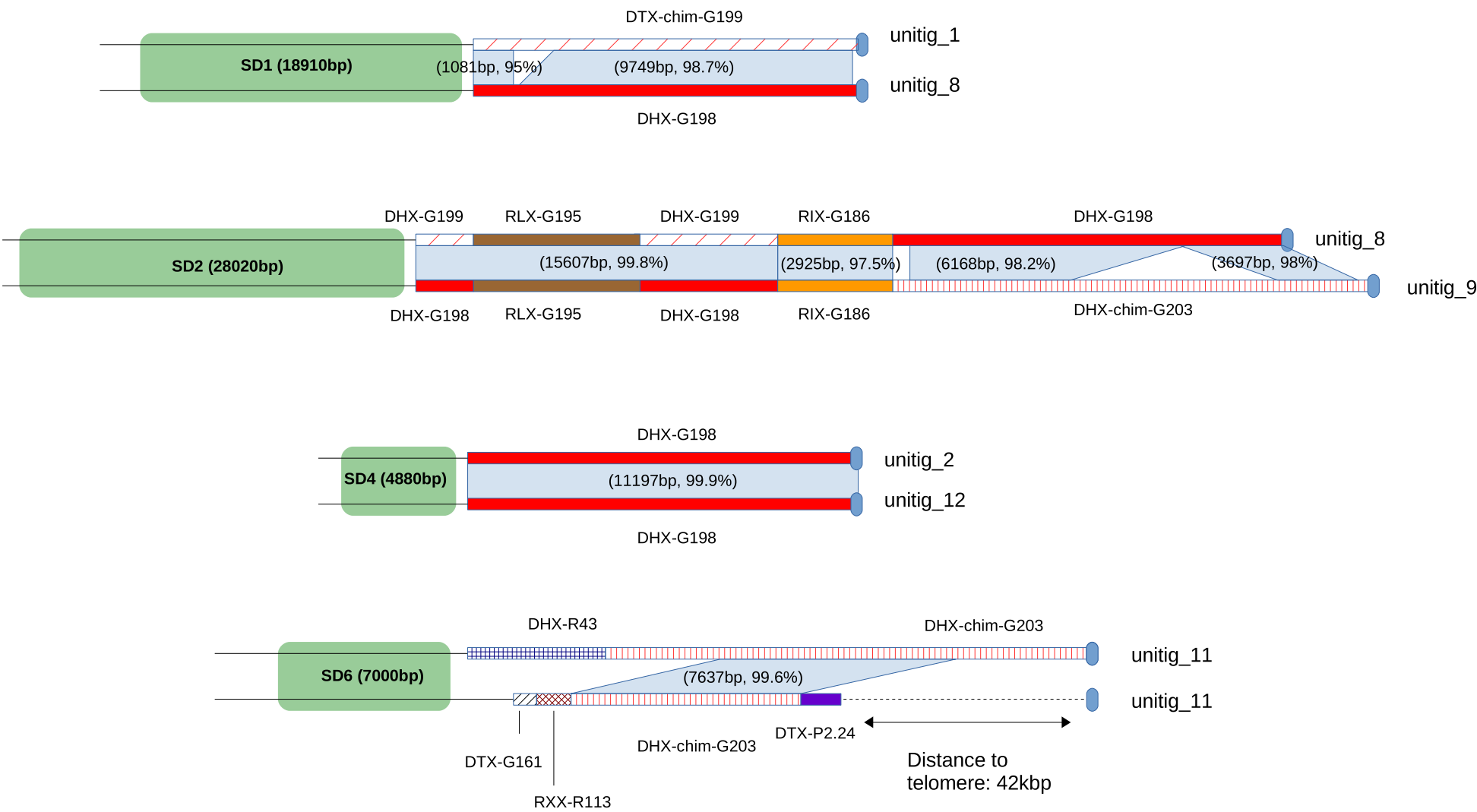

Supplement: Supplementary file 26 — Schematic representation of the association between four segmental duplications (shown in green) with the C. higginsianum subtelomeric repeats DHX_G198, DTX_chim-G199, and DHX_chim-G203. Homologous regions are shaded grey-blue. (PDF 747 kb) [file 12864_2017_4083_MOESM26_ESM.pdf]
