# Supplementary material for: Gapless genome assembly of Colletotrichum higginsianum reveals chromosome structure and association of transposable elements with secondary metabolite gene clusters
Source: BMC Genomics. 2017 Aug 29;18:667. doi: 10.1186/s12864-017-4083-x (PMC5576322; doi:10.1186/s12864-017-4083-x)
Supplement: Supplementary file 6 — Characteristics and contents of the 12 largest unitigs in the new genome assembly, corresponding to the 12 chromosomes of C. higginsianum. (PDF 129 kb) [file 12864_2017_4083_MOESM6_ESM.pdf]

Additional file 6: Characteristics and contents of the 12 largest unitigs in the new genome assembly, corresponding to the 12 chromosomes of *C. higginsianum*

Unitig	1	2	3	4	5	6	7	8	9	10	11	12
Length (bp)	6,042,495	6,014,962	5,992,392	5,325,643	5,201,688	4,523,820	4,439,061	4,422,509	4,092,876	3,084,912	646,208	597,935
No. of aligned old contigs*	1,062	1,106	1,088	967	984	809	801	773	742	540	108	2
Coverage by old contigs* (%)	88	88	88	86	88	85	88	85	84	85	73	1
No. of protein-coding genes	1,777	1,769	1,801	1,595	1,478	1,278	1,321	1,262	1,162	937	138	133
Total length of genes (bp)	2,828,823	2,782,298	2,906,176	2,466,936	2,341,721	1,973,624	2,036,068	1,939,891	1,822,983	1,525,193	164,739	152,101
Proportion of genes by length (%)	46.8	46.3	48.5	46.3	45.0	43.6	45.9	43.9	44.5	49.4	25.5	25.4
No. expressed genes†	1,024	991	1,073	873	831	646	707	670	620	431	44	13
Proportion (%) of expressed genes†	57.6	56.0	59.6	54.7	56.2	50.5	53.5	53.1	53.4	46.0	31.9	9.8
No. of TE copies	104	119	133	143	106	160	110	200	115	94	146	63
Total length of TEs (bp)	227,315	247,799	275,751	306,573	226,352	318,340	253,291	458,759	290,729	185,509	247,973	167,448
Proportion of TEs by length (%)	3.7	4.1	4.6	5.8	4.4	7.0	5.7	10.4	7.1	6.0	38.4	28.0
G+C (%)	54.7	55.4	54.9	54.4	55.1	54.2	54.7	53.0	54.6	54.2	49.3	47.2
Genes of unknown function	436	479	407	429	385	347	359	321	312	223	77	98
Proportion unknown function genes (%)	24.5	27.1	22.6	26.9	26.0	27.1	27.2	25.4	26.8	23.8	55.8	73.7
Genes encoding secreted proteins	205	167	205	202	164	129	129	148	140	121	14	10
Proportion secreted protein genes (%)	11.5	9.0	11.3	12.6	11.1	10.1	9.7	11.7	12.0	12.9	10.1	7.5
Effector (CSEP plus ChEC) genes#	35	38	32	30	25	29	14	20	26	20	8	6
Proportion effector genes (%)	1.96	2.14	1.78	1.88	1.69	2.27	1.06	1.58	2.24	2.13	5.79	4.51
SM key genes	5	7	6	11	10	7	5	10	12	16	0	1
CAZyme genes	99	70	79	89	67	58	58	68	53	57	1	1
Secreted protease genes	13	18	17	21	17	12	14	14	14	16	0	0
Transcription factor (TF) genes	75	83	92	64	67	68	64	58	43	51	1	2
P450 genes	26	25	25	24	24	14	19	30	21	32	0	1

*Alignment of 10,269 contigs from the old assembly (CACQ02000000) to the 12 largest unitigs (chromosomes) of the new assembly (LTAN00000000).

†Genes were considered to be expressed if they showed ≥1% of the expression-level of actin (corresponding to ≥10 TPM), based RNA-Seq data from one *in vitro* and three *in planta* samples (O'Connell et al. 2012).

#Candidate secreted effector protein genes included CSEPs predicted from the genome (secreted proteins without homologs outside the genus *Colletotrichum*) and some ChECs (*C. higginsianum* effector candidates) predicted from the transcriptome (Kleemann et al. 2012) that are absent from the new annotation or have BLAST hits to effectors from outside the genus.
