# Supplementary material for: Gapless genome assembly of Colletotrichum higginsianum reveals chromosome structure and association of transposable elements with secondary metabolite gene clusters
Source: BMC Genomics. 2017 Aug 29;18:667. doi: 10.1186/s12864-017-4083-x (PMC5576322; doi:10.1186/s12864-017-4083-x)
Supplement: Supplementary file 9 — List of the secondary metabolism key genes and their catalytic domains. (PDF 132 kb) [file 12864_2017_4083_MOESM9_ESM.pdf]

Additional File9: List of the secondary metabolism key genes and their catalytic domains.

Gene Name[a]	Protein ID	Cluster No.[b]	Domain struture (5' to 3')[c]	Previous Name(s)[d]	Notes
ChDMATS01	CH63R_03950	12	Trp_DMAT (IPR017795)	–	No Broad gene call.
ChDMATS02	CH63R_07283	25	Trp_DMAT (IPR017795)	DMATS08	
ChDMATS03	CH63R_07491	27	Trp_DMAT (IPR017795)	DMATS06	
ChDMATS04	CH63R_08007	30	Trp_DMAT (IPR017795)	DMATS09	
ChDMATS05	CH63R_09548	37	Trp_DMAT (IPR017795)	DMATS04	
ChDMATS06	CH63R_11302	46	Trp_DMAT (IPR017795)	DMATS03	
ChDMATS07	CH63R_12104	49	Trp_DMAT (IPR017795)	DMATS02	
ChDMATS08	CH63R_12343	53	Trp_DMAT (IPR017795)	DMATS01	
ChDMATS09	CH63R_13173	58	Trp_DMAT (IPR017795)	DMATS07	
ChDMATS10	CH63R_14290	72	Trp_DMAT (IPR017795)	DMATS10	
ChDMATS11	CH63R_14373	75	Trp_DMAT (IPR017795)	DMATS05	
ChNRPS01	CH63R_01925	05	A-PCP-C-A-PCP-C-A-MT-PCP-C-A-PCP-C-A-MT-PCP-C-A-PCP-C-A-PCP-C-A-PCP-C-A-PCP-C-A-MT-PCP-C	NRPS02	
ChNRPS02	CH63R_02472	07	A-PCP-C-A-PCP-C-A-PCP-C- A-PCP-C-A-PCP-C-A-PCP-C-PCP-E-C-A-PCP-C	NRPS08	
ChNRPS03	CH63R_03546	09	E-C-A-PCP	–	CH063_12860
ChNRPS04	CH63R_07082	24	A-PCP-C	NRPS13	
ChNRPS05	CH63R_08460	32	A-C-A-PCP-C-PCP-C-A-PCP-C-PCP-C-C	NRPS03	
ChNRPS06	CH63R_08655	33	A-PCP-C-A-PCP-C	NRPS06	
ChNRPS07	CH63R_09537	36	A-PCP-C-A-PCP-C-A-PCP-C-A-PCP-C	NRPS04	
ChNRPS08	CH63R_10509	43	A-PCP-C-A-Red	NRPS07	
ChNRPS09	CH63R_12341	53	C-A-PCP-C-A-PCP-C-A-PCP-E-C-A-PCP-C	NRPS09	
ChNRPS10	CH63R_12355	54	PCP-E-C-A-PCP-C	NRPS14	
ChNRPS11	CH63R_12697	57	A-PCP-C-PCP-C	NRPS12	
ChNRPS12	CH63R_13267	61	C-A-PCP-E-C-A-PCP-C	NRPS11	
ChNRPS13	CH63R_13518	63	A-PCP-C-A-Red	NRPS10	
ChNRPS14	CH63R_13536	64	C-A-PCP-C-A-PCP-C	–	CH063_08287
ChNRPS15	CH63R_14246	69	A-PCP-C	NRPS05	
ChNRPS-like01	CH63R_05219	13	A-PCP-Red	–	
ChNRPS-like02	CH63R_05229	13	A-PCP-Red	–	
ChNRPS-like03	CH63R_05403	15	A	–	
ChNRPS-like04	CH63R_05470	16	A-PCP-Red	–	
ChNRPS-like05	CH63R_06618	22	A-PCP-Red	–	
ChNRPS-like06	CH63R_06645	23	A-PCP-Red-D	NRPS01	
ChNRPS-like07	CH63R_07605	29	A-PCP-Red	–	
ChNRPS-like08	CH63R_09612	38	A-PCP-Red	–	
ChNRPS-like09	CH63R_09740	40	A-PCP-Red	–	
ChNRPS-like10	CH63R_10366	42	A-PCP-Red	–	
ChNRPS-like11	CH63R_14253	70	A-PCP	–	
ChNRPS-like12	CH63R_14351	74	A-Red	–	
ChPKS01	CH63R_00144	01	KS	PKS30	Disrupted by TEs.
	CH63R_00145	01	AT-DH-MT-KR-ACP	PKS3	
ChPKS02	CH63R_00148	01	KS-AT-DH-KR-ACP	PKS4	
ChPKS03	CH63R_01563	03	KS-AT-DH-MT-ER-KR-ACP	PKS55 + PKS31	
ChPKS04	CH63R_01782	04	KS-AT-DH-MT-ER-KR-ACP	PKS38	
ChPKS05	CH63R_02178	06	KR-ACP	PKS48	Fgenesh predicts a gene including CH63R_02180, CH63R_02179 and CH63R_02178 with the following domains: KS-DH-ER-KR-ACP.
	CH63R_02179	06	ER	PKS54	
	CH63R_02180	06	KS	PKS50	
ChPKS06	CH63R_03559	10	KS-AT-DH-ER-KR-ACP	PKS33	
ChPKS07	CH63R_03560	10	SAT-KS-AT-PT-ACP-TE	PKS47	
ChPKS08	CH63R_03864	11	SAT-KS-AT-PT-ACP-MT	PKS16	
ChPKS09	CH63R_03958	12	KS-AT-DH-ER-KR	PKS28	
ChPKS10	CH63R_05469	16	KS-AT-DH-ACP	PKS23	
ChPKS11	CH63R_05473	16	SAT-KS-AT-PT-ACP-MT	PKS42 + PKS52	
ChPKS12	CH63R_06245	18	SAT-KS-AT-PT-ACP-TE/CLC	PKS13	
ChPKS13	CH63R_06316	19	KS-AT-DH-ER-KR-ACP	PKS2	
ChPKS14	CH63R_06535	21	KS-AT-DH-ER-KR-ACP	PKS37 + PKS53	
ChPKS15	CH63R_07083	24	KS-AT-DH-MT-ER-KR-ACP	PKS49 + PKS56	
ChPKS16	CH63R_07287	25	SAT-KS-AT-PT-ACP-TE/CLC	PKS46	
ChPKS17	CH63R_07577	28	KS-AT-DH-ER-KR	PKS32	
ChPKS18	CH63R_08368	31	KS-AT-DH-KR-ACP	PKS34	
ChPKS19	CH63R_08918	34	SAT-KS-AT-PT-ACP-ACP-TE	PKS17	Melanin
ChPKS20	CH63R_09230	35	KS-AT-DH-ER-KR-ACP	PKS45	
ChPKS21	CH63R_09687	39	KS-AT-DH-MT-KR-ACP	PKS22 + PKS24	
ChPKS22	CH63R_09713	–	AT-DH-MT-KR	PKS21	Lack a KS domain. TEs at 3'end.
ChPKS23	CH63R_10337	41	KS-AT-DH-MT-ER-KR-ACP	PKS20	
ChPKS24	CH63R_10649	44	KS-AT-DH-MT-ER-KR-ACP	PKS10	
ChPKS25	CH63R_11486	47	KS-AT-DH-MT-ER-KR-ACP	PKS54	
ChPKS26	CH63R_12222	50	SAT- KS-AT-PT-ACP-MT-Red	PKS12	
ChPKS27	CH63R_12276	52	KS-AT-DH-ACP-MT	–	No Broad gene call.
ChPKS28	CH63R_12368	55	KS-AT-DH-KR-ACP	PKS14	
ChPKS29	CH63R_13251	60	KS-AT-DH-MT-ER-KR-ACP	PKS06	
ChPKS30	CH63R_13259	60	KS-AT-DH-KR-ACP	PKS15	
ChPKS31	CH63R_13453	62	KS-AT-DH-MT-ER-KR-ACP	PKS25 + PKS29	
ChPKS32	CH63R_13790	65	KS-AT-DH-MT-ER-KR-ACP	PKS35	
ChPKS33	CH63R_13915	66	KS-AT-DH-ER-KR-ACP	PKS09	
ChPKS34	CH63R_13918	66	KS	PKS08	Disrupted by TEs.

Gene Name[a]	Protein ID	Cluster No.[b]	Domain struture (5' to 3')[c]	Previous Name(s)[d]	Notes
	CH63R_13919	66	AT-DH-MT-ER-KR-ACP	PKS08	
ChPKS35	CH63R_14003	67	KS-AT-DH-ER-KR	PKS36 + PKS39 + PKS44	
ChPKS36	CH63R_14317	73	KS-AT-DH-MT-ER-KR-ACP	PKS05	
ChPKS37	CH63R_14349	74	KS-AT-DH-ER-KR-ACP	PKS19	
ChPKS38	CH63R_14350	74	SAT-KS-AT-PT-ACP-ACP-TE	PKS18	
ChPKS39	CH63R_14371	75	SAT-KS-AT-PT-ACP-MT	PKS27 + PKS51	
ChPKS40	CH63R_14522	76	KS-AT-DH-MT-ER-KR-ACP	–	No Broad gene call.
ChPKS type III	CH63R_06424	–	Chalcone synthase-like	–	
ChPKS-NRPS01	CH63R_01271	02	KS-AT-DH-MT-KR-C-A-PCP-Red	PKS-NRPS03	
ChPKS-NRPS02	CH63R_03382	08	KS-AT-DH-MT-KR-C-A-PCP-Red	PKS-NRPS01	Ace1 homolog.
ChPKS-NRPS03	CH63R_05344	14	KS-AT-DH-MT-KR-C-A-PCP-Red	PKS-NRPS04	Syn2 homolog.
ChPKS-NRPS04	CH63R_10646	44	KS-AT-DH-MT-KR-ACP-C	PKS11	
ChPKS-NRPS05	CH63R_14031	68	KS-AT-DH-MT-KR-C-A-PCP-Red	PKS-NRPS06	
ChPKS-NRPS06	CH63R_14283	71	KS-AT-DH-MT-KR-ACP-C	PKS41 / PKS-NRPS05	
ChTS01	CH63R_01285	–	Terpenoid synthases (IPR008949)	TS14	Wrong gene model: Fgenesh-Colletotrichum model predict a different gene. The resulting protein has the two motifs corresponding to TS.
ChTS02	CH63R_01882	–	Terpenoid synthases (IPR008949)	–	No Broad gene call.
ChTS03	CH63R_05405	15	Terpenoid synthases (IPR008949)	TS10	
ChTS04	CH63R_05999	–	Terpenoid synthases (IPR008949)	TS17	
ChTS05	CH63R_06219	17	Terpenoid synthases (IPR008949)	TS08	
ChTS06	CH63R_06464	20	Terpenoid synthases (IPR008949) - Polyprenyl_synth (IPR000092)	TS01	Putative diterpene cyclase
ChTS07	CH63R_06527	21	Terpenoid synthases (IPR008949)	–	No Broad gene call.
ChTS08	CH63R_07462	26	Terpenoid synthases (IPR008949)	TS06	Carotenoid
ChTS09	CH63R_08252	–	Terpenoid synthases (IPR008949)	–	No Broad gene call.
ChTS10A	CH63R_11027	45A	Terpenoid synthases (IPR008949)	TS12	
ChTS11	CH63R_11299	46	Terpenoid synthases (IPR008949) - Polyprenyl_synth (IPR000092)	TS16	Putative diterpene cyclase
ChTS12	CH63R_11724	48	Terpenoid synthases (IPR008949)	TS13	
ChTS13	CH63R_12251	51	Terpenoid synthases (IPR008949) - Polyprenyl_synth (IPR000092)	TS03	Putative diterpene cyclase
ChTS14	CH63R_12275	52	Terpenoid synthases (IPR008949)	–	No Broad gene call.
ChTS10B	CH63R_12288	45B	Terpenoid synthases (IPR008949)	TS12	
ChTS15	CH63R_12498	56	Terpenoid synthases (IPR008949) - Polyprenyl_synth (IPR000092)	TS09	Putative diterpene cyclase
ChTS16	CH63R_13231	59	Terpenoid synthases (IPR008949) - Polyprenyl_synth (IPR000092)	TS05	Putative diterpene cyclase

[a,b] This study.

[c] Determined using InterProScan v5 (Jones et al., 2014) and antiSMASH v3 (Weber et al., 2015). SAT: starter unit acyltransferase; KS: ketoacyl synthase; AT: acyl transferase; DH: dehydratase; PT: product template; ER: enoyl reductase; KR: ketoacyl reductase; MT: methyl transferase; ACP: acyl carrier protein; TE: thioesterase; A: adenylation; PCP: peptidyl carrier protein; C: condensation; E: epimerization; Red: reductase; D: short-chain dehydrogenase/reductase.

[d] As published by O'Connell *et al*. 2012.
