# Supplementary material for: Gapless genome assembly of Colletotrichum higginsianum reveals chromosome structure and association of transposable elements with secondary metabolite gene clusters
Source: BMC Genomics. 2017 Aug 29;18:667. doi: 10.1186/s12864-017-4083-x (PMC5576322; doi:10.1186/s12864-017-4083-x)
Supplement: Supplementary file 13 — Size distribution of Simple Sequence Repeats (SSR) in the C. higginsianum genome. (PDF 245 kb) [file 12864_2017_4083_MOESM13_ESM.pdf]

Additional file 13: Size distribution of Simple Sequence Repeats (SSR) in the *Colletotrichum higginsianum* genome

SSR motif length	Matches not merged		After merging overlapping annotations	
	**No. matches**	**Total length (bp)**	**No. matches**	**Total length (bp)**
1 nt	11,134	339,739	10,671	328,796
2 nt	11,414	420,551	10,361	381,391
3 nt	34,676	1,132,185	33,195	1,074,234
4 nt	26,119	558,586	25,602	541,020
5 nt	25,385	518,971	24,989	506,361
6 nt	36,702	915,762	36,497	907,701
7 nt	13,650	355,403	13,641	355,403
8 nt	10,354	311,258	10,350	311,258
9 nt	16,162	550,302	16,152	550,302
10 nt	7,798	251,541	7,796	251,541
11 nt	6,163	225,039	6,162	225,039
12 nt	9,274	393,326	9,268	393,264
13 nt	4,194	176,627	4,193	176,627
14 nt	3,307	154,673	3,307	154,673
15 nt	4,369	222,236	4,369	222,236
16 nt	637	29,468	637	29,468
17 nt	99	5,293	99	5,293
18 nt	29	1,470	29	1,470
19 nt	1	54	1	54
**Total**	**221,467**	**6,562,484**	**217,319**	**6,416,131**
