# Supplementary material for: Gapless genome assembly of Colletotrichum higginsianum reveals chromosome structure and association of transposable elements with secondary metabolite gene clusters
Source: BMC Genomics. 2017 Aug 29;18:667. doi: 10.1186/s12864-017-4083-x (PMC5576322; doi:10.1186/s12864-017-4083-x)
Supplement: Supplementary file 14 — Genome content and characteristics of 41 transposable element families in C. higginsianum. (PDF 521 kb) [file 12864_2017_4083_MOESM14_ESM.pdf]

Additional file 14: Genome content and characteristics of 41 transposable element families in *Colletotrichum higginsianum*

Transposable element concensus sequence ID[a]	Length (bp)	Genome coverage (bp)	No. fragments	No. full length fragments	No. copies	No. full length copies	Mean identity to consensus (%)	Mean length of consensus (bp)	Mean length as percent of consensus	AT richness (%)
Class I (retrotransposon)	*7,354.35*	*2,383,078.00*	*990*	*101*	*856*	*111*	*84.82*	*2,581.04*	*34.99*	*57.0*
**Long terminal repeat (LTR)**										
Ccret1_EF067893[b]	1,784	10,467	14	1	9	1	69.40	1163.00	65.19	46.0
RLX_G190	7,554	32,153	16	3	15	3	89.70	2196.00	29.07	49.2
RLX_G195	7,550	121,750	28	13	26	14	91.03	4694.77	62.18	48.6
RLX_G196	7,527	34,116	22	5	19	5	94.79	2258.89	30.01	49.0
RLX-chim_R14	6,848	45,831	21	3	16	4	93.65	2878.69	42.04	71.5
RLX_G189	6,183	61,562	80	7	75	8	82.38	849.75	13.74	42.9
RLX_P25.13	7,637	25,916	11	3	11	3	95.08	2404.27	31.48	48.8
RLX_R119	7,987	1,006,334	331	34	275	35	79.53	3673.51	45.99	78.0
RLX-R5	5,829	47,304	54	1	48	1	79.54	985.50	16.91	73.6
RLX_R58	7,625	261,532	92	6	72	10	79.67	3745.62	49.12	75.4
RLX_R71	7,645	156,939	77	1	70	2	80.96	2241.99	29.33	75.2
**Large retrotransposon derivative element (LARD)**										
RXX-LARD_G201	19,339	81,154	23	1	22	1	87.39	3694.82	19.11	48.8
RXX-LARD_R1	20,366	258,272	25	9	25	9	97.12	10474.48	51.43	48.6
**Long interspersed element (LINE)**										
RIX_P38.1	5,938	31,960	27	3	20	4	81.85	1600.90	26.96	47.6
RIX_G186	6,126	62,279	24	6	21	6	94.56	2966.43	48.42	43.6
RIX_P24.14	5,846	22,465	9	3	9	3	90.72	2496.11	42.70	43.7
**Class I (unclassified)**										
Ccret3_EF067892[b]	1,556	2,743	8	0	6	0	79.73	457.17	29.38	48.3
CgT1_L76205[c]	5,812	12,895	15	0	13	0	69.98	993.00	17.09	48.6
RXX_R113	2,421	15,254	24	1	23	1	79.85	695.35	28.72	77.5
RXX_R62	5,514	92,152	89	1	81	1	79.38	1150.52	20.87	76.0
Class II (DNA transposon)	*3,825.95*	*1,168,278*	*639*	*321*	*615*	*325*	*90.75*	*1,677.69*	*67.60*	*52.8*
**Terminal inverted repeat (TIR)**										
DTX_G157	1,875	10,389	7	3	7	3	94.66	1,484.14	79.15	50.9
DTX_G160	1,898	20,289	15	10	15	10	92.97	1,352.60	71.26	50.5
DTX_G161	1,913	62,393	39	31	38	31	95.83	1,641.92	85.83	50.6
DTX_G164	1,887	47,033	31	23	29	24	95.70	1,621.83	85.95	49.9
DTX_P12.24	1,925	52,458	34	25	32	25	96.15	1,639.31	85.16	50.0
DTX_P2.24	1,894	62,026	42	29	39	31	95.31	1,590.41	83.97	50.4
DTX_P21.16	1,892	19,148	11	10	11	10	97.58	1,740.73	92.00	50.4
DTX_P40.29	1,863	13,295	18	5	15	5	83.66	887.33	47.63	50.9
DTX_R12	1,868	14,940	10	6	10	6	91.58	1,494.00	79.98	51.3
DTX_R31	1,871	152,513	85	80	84	81	99.05	1,815.63	97.04	51.1
DTX-chim_G199	12,831	259,659	109	7	105	7	81.56	2,472.94	19.27	42.4
DTX_G154	1,949	43,632	25	16	25	16	87.20	1,745.28	89.55	52.8
DTX_G156	1,922	55,376	33	25	33	25	91.78	1,678.06	87.31	53.1
DTX_P20.17	832	10,167	15	9	15	9	94.01	677.80	81.47	77.6
DTX_R124	1,498	5,670	11	2	11	2	83.96	515.45	34.41	71.4
DTX_R166	1,829	5,613	5	4	5	4	95.96	1,473.60	80.57	49.5
**Miniature inverted-repeat transposable element (MITE)**										
DXX-MITE_G118	852	19,148	30	17	30	17	88.33	638.27	74.91	75.5
**Helitron**										
DHX-chim_G203	18,549	118,203	41	4	39	4	82.07	3,030.97	16.34	44.2
DHX_G198	11,268	160,658	34	12	31	12	87.96	5,182.52	45.99	41.5
DHX_R43	6,103	35,668	44	3	41	3	79.76	870.95	14.27	41.0
Uncategorized TEs										
noCat_G49	494	4,474	11	4	11	4	96.59	406.73	82.33	68.6

[a] Transposable elements (TE) were analyzed using the REPET pipeline (Flutre et al 2011). The classification of TEs uses the three-letter code described by Wicker et al. (2007). (R, retrotransposon; D, DNA transposon), order (L, Long terminal repeat—LTR; T, terminal inverted repeat—TIR;) and superfamily (G, Gypsy; C, Copia; I, LINE; T, Tc1-Mariner; H, Helitron; X, unknown superfamily). The suffix 'chim' indicates a chimeric TE.

[b] Transposable elements reported from *C. cereale* (Crouch et al. 2007). Suffixes represent their respective GenBank accession numbers.

[c] Transposable element reported from *C. gloeosporioides* (He et al. 1996). Suffix represents the GenBank accession number.
