# Supplementary material for: Gapless genome assembly of Colletotrichum higginsianum reveals chromosome structure and association of transposable elements with secondary metabolite gene clusters
Source: BMC Genomics. 2017 Aug 29;18:667. doi: 10.1186/s12864-017-4083-x (PMC5576322; doi:10.1186/s12864-017-4083-x)
Supplement: Supplementary file 17 — Plot showing the distribution of C. higginsianum transposable element families across the 25 largest unitigs. (PDF 193 kb) [file 12864_2017_4083_MOESM17_ESM.pdf]

Additional file 17

TE Families	unitigs																							
	1	2	3	4	5	6	7	8	9	10	11	12	13	14	15	16	17	18	19	20	21	22	23	24	25
DTX-R31	■	■	■	■	■	■	■	■	■	■	■	■													
RLX-R119	■	■	■	■	■	■	■	■	■	■	■	■													
DTX-G161	■	■	■	■	■	■	■	■	■	■	■														
DTX-G164	■		■	■	■	■	■	■	■	■	■	■													
DTX-P2.24		■	■	■	■	■	■	■	■	■	■	■													
RLX-R58	■	■	■	■		■	■	■	■	■	■														
DTX-G154	■	■	■	■	■	■	■	■	■		■														
DTX-G156	■	■	■	■	■	■	■	■	■			■													
DHX-G198	■	■	■	■	■	■	■	■				■													
DTX-P12.24		■	■	■	■	■		■	■		■	■													
DXX-MITE-G118	■	■		■	■	■	■	■		■															
RLX-G195-Map11	■		■	■		■	■	■	■	■		■													
DTX-G160-Map10	■	■		■	■	■			■		■	■													
DTX-chim-G199	■	■			■	■				■		■													
DTX-P20.17	■	■		■		■			■		■														
RLX-R71	■	■	■			■	■					■													
DTX-P21.16		■	■			■		■		■															
DTX-R12		■		■				■	■	■															
RIX-G186					■	■	■		■			■													
RLX-G189		■			■		■		■	■															
RLX-G196	■	■				■	■																		
noCat-G49	■	■					■		■																
RLX-chim-R14	■	■					■			■															
DTX-P40.29	■		■			■					■														
RLX-P25.13									■	■		■													
CCRET1_EF067893:ClassI:LTR:_						■	■				■														
DHX-chim-G203									■		■		■												
DHX-R43					■		■				■														
RLX-G190		■				■			■																
DTX-R124					■			■																	
RIX-P38.1					■						■														
DTX-R166					■						■														
RIX-P24.14											■	■													
RXX-R113		■																							
RXX-R62					■																				
RLX-R5							■																		
DTX-G157							■																		
RXX-LARD-G201							■																		
RXX-LARD-R1														■	■	■	■	■	■	■			■	■	
