# Supplementary material for: Gapless genome assembly of Colletotrichum higginsianum reveals chromosome structure and association of transposable elements with secondary metabolite gene clusters
Source: BMC Genomics. 2017 Aug 29;18:667. doi: 10.1186/s12864-017-4083-x (PMC5576322; doi:10.1186/s12864-017-4083-x)

Additional file 18

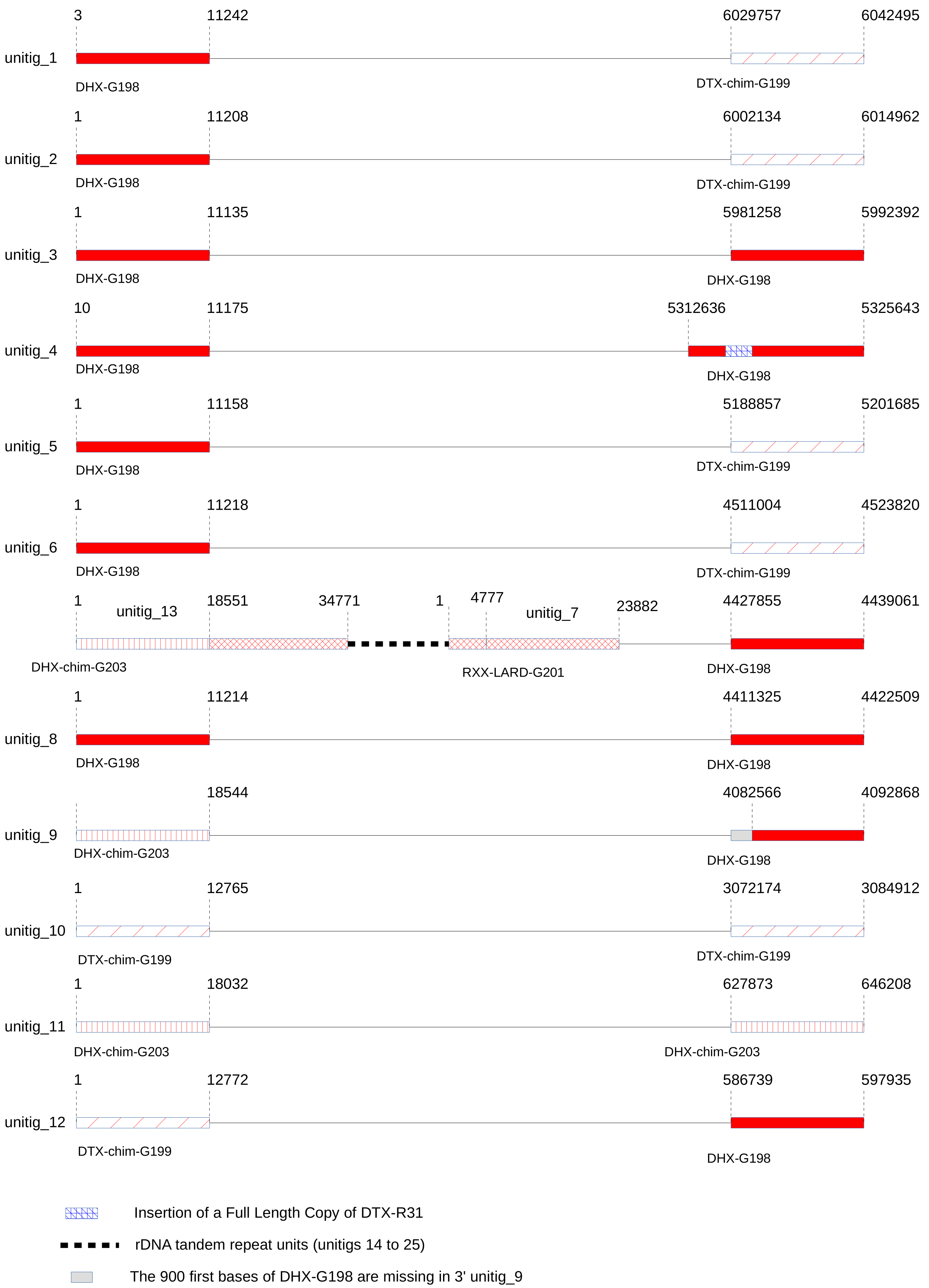

Supplement: Supplementary file 18 — Schematic representation of the distribution of three families of conserved repeats at the 24 subtelomeres of C. higginsianum. (PDF 1186 kb) [file 12864_2017_4083_MOESM18_ESM.pdf]
