# Supplementary material for: Gapless genome assembly of Colletotrichum higginsianum reveals chromosome structure and association of transposable elements with secondary metabolite gene clusters
Source: BMC Genomics. 2017 Aug 29;18:667. doi: 10.1186/s12864-017-4083-x (PMC5576322; doi:10.1186/s12864-017-4083-x)
Supplement: Supplementary file 20 — Expression of TE copies based on average log (CPM), with a threshold of 1.35. Results are displayed for uniquely-mapped and multi-mapped read counts. (PDF 299 kb) [file 12864_2017_4083_MOESM20_ESM.pdf]

Additional file 20: Expression of TE copies based on average log(CPM), with a threshold of 1.35. Results are displayed for unique-mapped and multi-mapped read counts.

TE Family	No. copies	Expressed TE copies		Percentage of TE family	
		unique	multi	unique	multi
CCRET1_EF067893	9	0	0	0,0	0,0
RLX_G190	15	3	5	20,0	33,3
RLX_G195	26	0	15	0,0	57,7
RLX_G196	19	1	3	5,3	15,8
RLX-chim_R14	16	0	0	0,0	0,0
RLX_G189	75	3	11	4,0	14,7
RLX_P25.13	11	2	6	18,2	54,5
RLX_R119	275	0	0	0,0	0,0
RLX_R5	48	0	0	0,0	0,0
RLX_R58	72	4	8	5,6	11,1
RLX_R71	70	0	0	0,0	0,0
RXX-LARD_G201	22	0	12	0,0	54,5
RXX-LARD_R1	25	1	19	4,0	76,0
RIX_P38.1	20	1	4	5,0	20,0
RIX_G186	21	0	12	0,0	57,1
RIX_P24.14	9	0	4	0,0	44,4
CCRET3_EF067892	6	0	0	0,0	0,0
COGNLRC_CGT1	13	1	3	7,7	23,1
RXX_R113	23	1	1	4,3	4,3
RXX_R62	81	0	0	0,0	0,0
DTX_G157	7	0	6	0,0	85,7
DTX_G160	15	1	11	6,7	73,3
DTX_G161	38	1	32	2,6	84,2
DTX_G164	29	3	24	10,3	82,8
DTX_P12.24	32	1	27	3,1	84,4
DTX_P2.24	39	2	2	5,1	5,1
DTX_P21.16	11	1	1	9,1	9,1
DTX_P40.29	15	0	0	0,0	0,0
DTX_R12	10	0	7	0,0	70,0
DTX_R31	84	1	80	1,2	95,2
DTX-chim_G199	105	1	81	1,0	77,1
DTX_G154	25	0	22	0,0	88,0
DTX_G156	33	0	28	0,0	84,8
DTX_P20.17	15	0	0	0,0	0,0
DTX_R124	11	0	0	0,0	0,0
DTX_R166	5	1	1	20,0	20,0
DXX-MITE_G118	30	0	0	0,0	0,0
DHX-chim_G203	39	2	9	5,1	23,1
DHX_G198	31	1	14	3,2	45,2
DHX_R43	41	0	0	0,0	0,0
noCat_G49	11	0	0	0,0	0,0
