# Supplementary material for: Gapless genome assembly of Colletotrichum higginsianum reveals chromosome structure and association of transposable elements with secondary metabolite gene clusters
Source: BMC Genomics. 2017 Aug 29;18:667. doi: 10.1186/s12864-017-4083-x (PMC5576322; doi:10.1186/s12864-017-4083-x)
Supplement: Supplementary file 22 — Genomic locations of TE copies from clusters 2 and 3 showing extreme expression profiles. (PDF 222 kb) [file 12864_2017_4083_MOESM22_ESM.pdf]

Additional file 22: Genomic locations of TE copies from clusters 2 and 3 showing extreme expression profiles

Cluster	TE family	Location	Start	End	Remarks
2	RLX_ G189	unitig_4	5309111	5309389	Located in intron of CH63R_06941. Likely a bad gene structure
2	RLX_ P25.13	unitig_9	785328	785518	-
3	RLX_ G189	unitig_6	4191021	4191273	Solo-LTR located in 3' UTR of CH63R _15028 (effector ChEC 28)
3	RLX_ G189	unitig_9	882325	882658	Located in intron of CH63R_12509. Likely a bad gene structure
3	RLX_ P25.13	unitig_6	4028071	4028644	Solo-LTR located in 3' UTR of CH63R _15029 (effector ChEC 117)
3	RLX_ P25.13	unitig_8	812305	812839	-
3	RLX_ R58	unitig_2	447678	454848	Located in 3' UTR of CH63R_01904 (effector ChEC 104)
3	RXX_ R113	unitig_2	25422	25620	Located in 3' UTR of CH63R _15012 (effector ChEC 35)
