# Supplementary material for: Gapless genome assembly of Colletotrichum higginsianum reveals chromosome structure and association of transposable elements with secondary metabolite gene clusters
Source: BMC Genomics. 2017 Aug 29;18:667. doi: 10.1186/s12864-017-4083-x (PMC5576322; doi:10.1186/s12864-017-4083-x)
Supplement: Supplementary file 23 — Results of permutation tests for the association of transposon families with secondary metabolism and effector genes. (PDF 363 kb) [file 12864_2017_4083_MOESM23_ESM.pdf]

Additional file 23: Results of permutation tests for the association of transposon families with secondary metabolism and effector genes

	**SM cluster genes**		**Effector genes**	
**TE family**	***p*-value**	***z*-score**	***p*-value**	***z*-score**
**Class I (retrotransposons)**				
Ccret1			3.0E-03	-2.73
RLX_G195	1.0E-04	-6.36		
RLX_G189	1.0E-04	-7.07	3.0E-03	-3.00
RLX_P25.13				
RLX_R119	1.0E-04	-4.50	9.0E-03	-2.21
RLX-R5	1.0E-04	-5.01	2.0E-03	-3.13
RXX_R62	1.0E-04	-6.19	7.0E-03	-2.32
**Class II (DNA transposons)**				
DTX_G161	1.0E-04	-5.44		
DTX_P12.24			1.0E-03	-3.43
DTX_P2.24	1.0E-04	-4.72		
DTX_P21.16			9.0E-03	-2.35
DTX_P40.29			5.0E-03	-2.84
DTX_R31			1.0E-03	-3.57
DTX-chim_G199	1.0E-04	-5.97	1.0E-03	-3.80
DTX_P20.17			2.0E-03	-2.52
DXX-MITE_G118	1.0E-04	-4.40		
DHX-chim_G203	1.0E-04	-4.46		
DHX_G198			6.0E-03	-2.44
