# Supplementary material for: Gapless genome assembly of Colletotrichum higginsianum reveals chromosome structure and association of transposable elements with secondary metabolite gene clusters
Source: BMC Genomics. 2017 Aug 29;18:667. doi: 10.1186/s12864-017-4083-x (PMC5576322; doi:10.1186/s12864-017-4083-x)
Supplement: Supplementary file 24 — Characteristics of six segmental duplications identified in the C. higginsianum genome assembly. (PDF 257 kb) [file 12864_2017_4083_MOESM24_ESM.pdf]

Additional file 24: Characteristics of six segmental duplications identified in the *Colletotrichum higginsianum* genome assembly.

		SD1	SD2	SD3	SD4	SD5	SD6
seq1	Unitig ID	unitig_1	unitig_8	unitig_6	unitig_2	unitig_6	unitig_11
	Coordinates	6010863-6029759	30501-58517	2839706-2849519	11786-16672	4466884-4473621	26582-33594
	Aligned length (bp)	18,897	28,017	9,814	4,887	6,738	7,013
	Number of genes	4	11	2	1	2	3
seq2	Unitig ID	unitig_8	unitig_9	unitig_10	unitig_12	unitig_7	unitig_11
	Coordinates	4391356-4410272	34425-62445	2227427-2237239	581868-586741	2982561-2989314	584193-591205
	Aligned length (bp)	18,917	28,021	9,813	4,874	6,754	7,013
	Number of genes	4	10	3	1	2	3
% Identity		97.3	100	100	99.8	99.9	99.9
Number of alignments		1	1	1	1	1	1
Number of SNPs		495	0	0	9	6	7
Number of InDels (bases)		34	18	1	15	16	0

Gaps between duplicated fragments were not included in SNP / Indels counts.
