# Supplementary material for: Gapless genome assembly of Colletotrichum higginsianum reveals chromosome structure and association of transposable elements with secondary metabolite gene clusters
Source: BMC Genomics. 2017 Aug 29;18:667. doi: 10.1186/s12864-017-4083-x (PMC5576322; doi:10.1186/s12864-017-4083-x)
Supplement: Supplementary file 25 — Gene content of six segmental duplications identified in the C. higginsianum genome assembly and polymorphisms between the duplicated genes. (PDF 319 kb) [file 12864_2017_4083_MOESM25_ESM.pdf]

Additional file 25: Gene content of six segmental duplications identified in the *Colletotrichum higginsianum* genome assembly and polymorphisms between duplicated genes

Duplication	Gene (seq1)	Gene (seq2)	#SNPs	#InDels	Effect
SD1	CH63R_01774	CH63R_12278	148	0	Many amino acid changes, premature stop codon, split gene, mutation bias G->A, C->T , possible RIP
	CH63R_01775	CH63R_12279	56	0	
	CH63R_01776		90	1	
		CH63R_12280	46	1	
	CH63R_01777	CH63R_12281	7	0	
SD2	CH63R_11020	CH63R_12282	0	0	None
	CH63R_11021	CH63R_12283	0	0	
	CH63R_11022	CH63R_12284	0	0	
	CH63R_11023	None	NA	NA	4 indels on unitig_9, (DNA-Seq and RNA-Seq mapping suggests PacBio sequencing errors)
	CH63R_11024	CH63R_12285	0	0	None
	CH63R_11025	CH63R_12286	0	0	
	CH63R_11026	CH63R_12287	0	0	
	CH63R_11027	CH63R_12288	0	0	
	CH63R_11028	CH63R_12289	0	1	Altered gene structure (shown to be false positive, by PCR and re-sequencing)
	CH63R_11029	CH63R_12290	0	0	None
	CH63R_11030	CH63R_12291	0	0	None, different gene prediction (isoform differential expression ?)
SD3	CH63R_09248	CH63R_14153	0	0	None
	CH63R_09249	CH63R_14152<br>CH63R_14151	0	1	Split gene structure in two genes (indel supported by DNA-Seq and RNA-Seq mapping)
SD4	CH63R_01779	CH63R_14651	0	1	None (indel located in intron)
SD5	CH63R_09688	CH63R_10606	0	0	None
	CH63R_09689	CH63R_10607	0	0	
SD6	CH63R_14381	CH63R_14511	0	0	None
	CH63R_14382	CH63R_14510	0	0	
	CH63R_14383	CH63R_14509	0	0	
